# SARS-CoV-2 spike receptor-binding domain is internalized and promotes protein ISGylation in human induced pluripotent stem cell-derived cardiomyocytes

**DOI:** 10.1038/s41598-023-48084-7

**Published:** 2023-12-04

**Authors:** Shota Okuno, Shuichiro Higo, Takumi Kondo, Mikio Shiba, Satoshi Kameda, Hiroyuki Inoue, Tomoka Tabata, Shou Ogawa, Yu Morishita, Congcong Sun, Saki Ishino, Tomoyuki Honda, Shigeru Miyagawa, Yasushi Sakata

**Affiliations:** 1 Department of Cardiovascular Medicine, Osaka University Graduate School of Medicine, Suita, Osaka 565-0871 Japan; 2 Department of Medical Therapeutics for Heart Failure, Osaka University Graduate School of Medicine, Suita, Osaka 565-0871 Japan; 3 CoMIT Omics Center, Osaka University Graduate School of Medicine, Suita, Osaka 565-0871 Japan; 4 Department of Virology, Okayama University Graduate School of Medicine, Dentistry and Pharmaceutical Sciences, Kita-Ku, Okayama 700-8558 Japan; 5 Department of Virology, Faculty of Medicine, Dentistry and Pharmaceutical Sciences, Okayama University, Kita-Ku, Okayama 700-8558 Japan; 6 Department of Cardiovascular Surgery, Osaka University Graduate School of Medicine, Suita, Osaka 565-0871 Japan

**Keywords:** Cardiovascular diseases, Induced pluripotent stem cells

## Abstract

Although an increased risk of myocarditis has been observed after vaccination with mRNA encoding severe acute respiratory syndrome coronavirus 2 spike protein, its underlying mechanism has not been elucidated. This study investigated the direct effects of spike receptor-binding domain (S-RBD) on human cardiomyocytes differentiated from induced pluripotent stem cells (iPSC-CMs). Immunostaining experiments using *ACE2* wild-type (WT) and knockout (KO) iPSC-CMs treated with purified S-RBD demonstrated that S-RBD was bound to ACE2 and internalized into the subcellular space in the iPSC-CMs, depending on ACE2. Immunostaining combined with live cell imaging using a recombinant S-RBD fused to the superfolder GFP (S-RBD-sfGFP) demonstrated that S-RBD was bound to the cell membrane, co-localized with RAB5A, and then delivered from the endosomes to the lysosomes in iPSC-CMs. Quantitative PCR array analysis followed by single cell RNA sequence analysis clarified that S-RBD-sfGFP treatment significantly upregulated the NF-kβ pathway-related gene (*CXCL1*) in the differentiated non-cardiomyocytes, while upregulated interferon (IFN)-responsive genes (*IFI6*, *ISG15*, and *IFITM3*) in the matured cardiomyocytes. S-RBD-sfGFP treatment promoted protein ISGylation, an ISG15-mediated post-translational modification in *ACE2*-WT-iPSC-CMs, which was suppressed in *ACE2*-KO-iPSC-CMs. Our experimental study demonstrates that S-RBD is internalized through the endolysosomal pathway, which upregulates IFN-responsive genes and promotes ISGylation in the iPSC-CMs.

## Introduction

Coronavirus disease 2019 (COVID-19), caused by severe acute respiratory syndrome-coronavirus-2 (SARS-CoV-2), has been declared a pandemic and poses a serious threat to public health^1,2^. Vaccination is the cornerstone of pandemic control, and COVID-19 vaccines using various platforms, including a new mRNA vaccine, have been developed with an unprecedented speed^3,4^. COVID-19 mRNA vaccines encoding SARS-CoV-2 spike proteins exhibit remarkable effectiveness^5–7^. However, after COVID-19 vaccination began in the general population, numerous case reports of myocarditis after the administration of COVID-19 vaccines have been published^8–12^, and a significant association between myocarditis and mRNA vaccines has been reported in a few observational studies^13–17^. COVID-19 vaccine-associated myocarditis is an important issue.

However, the mechanisms underlying myocarditis after COVID-19 vaccination are poorly understood. Although several mechanisms, including molecular mimicry or T-cell involvement by adaptive immunity, have been proposed; one possible hypothesis is that free-floating spike protein or its subunits/peptide fragments after proteolytic cleavage directly affect the cardiomyocytes by changing the gene expression to activate innate immunity^18–20^. Circulating exosomes expressing spike protein were detected in vaccinated participants^21^ and the S1 subunit, which is the subunit of the spike protein containing the receptor-binding domain (RBD), responsible for binding to ACE2, was also detected^22^. Additionally, a recent report indicated that circulating free spike antigen was present in adolescents and young adults with myocarditis after mRNA vaccination, although extensive antibody profiling and T-cell responses were indistinguishable between patients with myocarditis and healthy controls^23^. Histopathological analyses of endomyocardial biopsies or autopsies from patients with acute myocarditis following COVID-19 vaccination have revealed spike proteins and spike receptor binding domain (S-RBD) in the cardiomyocytes of several patients^24,25^. These findings suggest that the spike protein or its subunits/peptide fragments reach the heart, where they may directly change the gene expression and trigger an innate immune response.

S-RBD is the major target and a common domain for various types of COVID-19 vaccines that interfere with viral receptor binding^6,26–28^. Some studies have revealed that S-RBDs can cause inflammation. S-RBD promoted the activation and maturation of human dendritic cells with the activation of NF-kβ pathway^29^. S-RBD also significantly aggravated lipopolysaccharide-induced acute lung injury in an in vivo mouse model through NF-kβ pathway^30,31^. However, the effects of S-RBD on the innate immune response of cardiomyocytes have not yet been elucidated.

This study aimed to investigate the direct effects of the SARS-CoV-2 S-RBD on human cardiomyocytes. Here, we demonstrated the internalization of S-RBD into the induced pluripotent stem cell-derived cardiomyocytes (iPSC-CMs) via ACE2 through the endolysosomal pathway. We assessed the direct effect of S-RBD on the innate immune response in iPSC-CMs using quantitative PCR array analysis followed by single-cell RNA sequence (scRNA-seq) analysis and found that S-RBD upregulated interferon (IFN)-responsive genes in mature cardiomyocytes. Furthermore, ISG15 mediated post-translational modification (ISGylation) was promoted in *ACE2*-wild-type (WT)-iPSC-CMs and suppressed in *ACE2*-knockout (KO)-iPSC-CMs.

## Results

### SARS-CoV-2 S-RBD/ACE2 complex is internalized into the human iPSC-CMs

**Figure 1:**
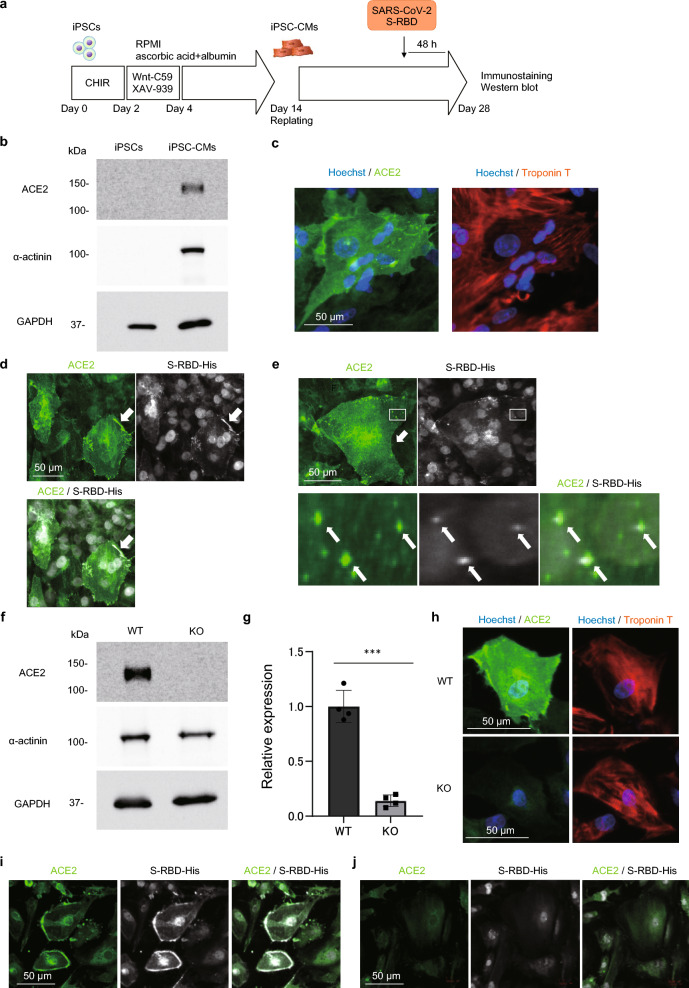
Internalization of SARS-CoV-2 S-RBD/ACE2 complex into the human iPSC-CMs. (**a**) Time course of monolayer differentiation into the cardiomyocytes. Differentiated cardiomyocytes were replated on day 14 after differentiation for further analysis. The iPSC-CMs were incubated with purified His-tagged SARS-CoV-2 S-RBD protein for 48 h before immunostaining and western blotting on day 28 after differentiation. (**b**)Whole cell lysates were extracted from the iPSCs and iPSC-CMs on day 28 after differentiation and analyzed by western blotting using the indicated antibodies. Original blots are presented in Supplementary Fig. S5. (**c**) The iPSC-CMs were fixed and immunostained with the indicated antibodies on day 28 after differentiation. Nuclei were detected by Hoechst staining. (**d**) The iPSC-CMs were incubated with 1250 ng/mL purified His-tagged SARS-CoV-2 S-RBD protein for 48 h before immunostaining on day 28 after differentiation with the indicated antibodies. White arrows show the accumulation of S-RBD at the periphery of the iPSC-CMs co-localized with ACE2. (**e**) The iPSC-CMs were incubated with 1250 ng/mL purified His-tagged SARS-CoV-2 S-RBD protein for 48 h before immunostaining on day 28 after differentiation with the indicated antibodies. Areas enclosed within the white squares are enlarged at the bottom. White arrows indicate the S-RBD/ACE2 complex internalized in the subcellular space. (**f**) Whole cell lysates were extracted from *ACE2*-WT-iPSC-CMs and *ACE2*-KO-iPSC-CMs on day 28 after differentiation and analyzed by western blotting using the indicated antibodies. Original blots are presented in Supplementary Fig. S5. (**g**) Quantified ACE2 protein expression levels were normalized by GAPDH expression in *ACE2*-WT-iPSC-CMs and *ACE2*-KO-iPSC-CMs (n = 4). Data are presented as the mean ± SD. Statistical differences were calculated using Student’s t-test. ***p < 0.001. (**h**) *ACE2*-WT-iPSC-CMs and *ACE2*-KO-iPSC-CMs were fixed and immunostained on day 28 after differentiation using the indicated antibodies. (**i**) *ACE2*-WT-iPSC-CMs were incubated with 1250 ng/mL purified His-tagged SARS-CoV-2 S-RBD protein for 48 h before immunostaining on day 28 after differentiation with the indicated antibodies. (**j**) *ACE2*-KO-iPSC-CMs were incubated with 1250 ng/mL purified His-tagged SARS-CoV-2 S-RBD protein for 48 h before immunostaining on day 28 after differentiation with the indicated antibodies.

We first addressed whether the human iPSC-CMs expressed ACE2, the primary receptor of SARS-CoV-2. We used the induced pluripotent stem cells (iPSCs) generated from a male patient with hypertrophic cardiomyopathy (HCM)^32^ and analyzed ACE2 expression using western blotting and immunostaining after monolayer differentiation (Fig. 1a). ACE2 protein was expressed in the iPSC-CMs but not in undifferentiated iPSCs (Fig. 1b) and was localized mainly at the cell membrane and perinuclear region and partly in the cytosol (Fig. 1c), consistent with previous reports^33,34^. ACE2 expression was not altered in the iPSC-CMs carrying the corrected mutation (Supplementary Fig. S1a, b) or in the iPSC-CMs generated from a female healthy donor (Supplementary Fig. S1c, d), suggesting that either the HCM mutation or gender difference did not significantly affect the protein expression of ACE2 in our experimental conditions. To evaluate whether SARS-CoV-2 S-RBD binds to the iPSC-CMs, the iPSC-CMs were incubated with purified His-tagged SARS-CoV-2 S-RBD protein, which contains the amino acids Arg 319–Phe 541 of the SARS-CoV-2 Spike protein, for 48 h. Immunostaining revealed that S-RBD accumulated at the periphery of the iPSC-CMs co-localized with ACE2 (Fig. 1d and Supplementary Fig. S1e) and was then internalized as the S-RBD/ACE2 complex in the subcellular space (Fig. 1e). Immunoprecipitation analysis further confirmed the binding of ACE2 and S-RBD (Supplementary Fig. S1f.). We generated an *ACE2* KO iPSC clone (*ACE2*-KO-iPSCs) carrying a frameshift mutation (c.290dupT) in exon 2 using genome editing (Supplementary Fig. S1g, h), which had uniformly round colonies, were positive for pluripotency markers, negative for virus-mediated transgenes, and exhibited normal karyotypes (Supplementary Fig. S1i-l). Western blotting and immunostaining analyses showed that the protein expression level of *ACE2* was abolished in *ACE2*-KO-iPSC-CMs compared to that in the original iPSC-CMs (*ACE2*-WT-iPSC-CMs) (Fig. 1f–h). After treatment with S-RBD for 48 h, S-RBD signals were detected at the cell membrane and co-localized with ACE2 in *ACE2*-WT-iPSC-CMs (Fig. 1i). In contrast, neither membrane binding nor internalization of S-RBD was detected in *ACE2-*KO-iPSC-CMs (Fig. 1j), suggesting that internalization of SARS-CoV-2 S-RBD depends on its binding to ACE2.

### SARS-CoV-2 S-RBD uses endolysosomal pathway internalizing into the iPSC-CMs

**Figure 2:**
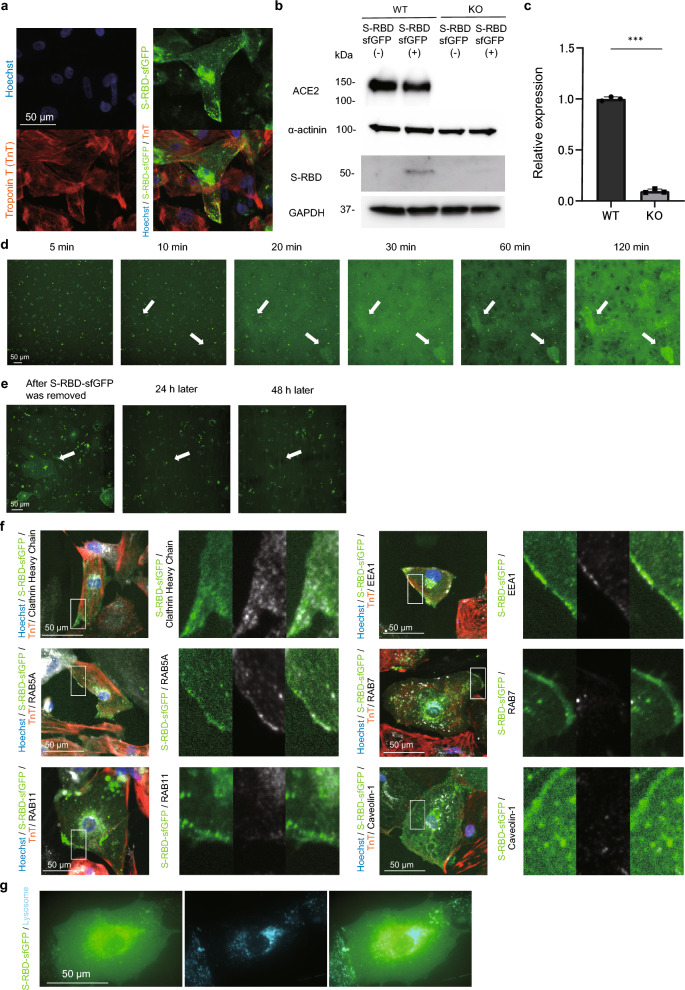
Internalization of SARS-CoV-2 S-RBD-sfGFP into the iPSC-CMs through endolysosomal pathway. (**a**) *ACE2*-WT-iPSC-CMs were incubated with SARS-CoV-2 S-RBD-sfGFP for 48 h before immunostaining on day 28 after differentiation with the indicated antibodies. (**b**) Whole cell lysates were extracted from *ACE2*-WT-iPSC-CMs and *ACE2*-KO-iPSC-CMs on day 28 after differentiation and analyzed by western blotting using the indicated antibodies. Both iPSC-CMs were incubated with 1,200 ng/mL SARS-CoV-2 S-RBD-sfGFP for 48 h prior to western blotting. Original blots are presented in Supplementary Fig. S5. (**c**) Quantified S-RBD-sfGFP expression levels were normalized by GAPDH expression in *ACE2*-WT-iPSC-CMs and *ACE2*-KO-iPSC-CMs (n = 3). Data are presented as the mean ± SD. Statistical differences were calculated using the Student’s t-test. ***p < 0.001. (**d**) Time-lapse imaging of *ACE2*-WT-iPSC-CMs after S-RBD-sfGFP treatment. S-RBD-sfGFP-positive iPSC-CMs were detected at approximately 10 min after S-RBD-sfGFP treatment (white arrows). (**e**) Time-lapse imaging of *ACE2*-WT-iPSC-CMs after S-RBD-sfGFP treatment. The S-RBD-sfGFP signals disappeared completely 48 h after the removal of S-RBD-sfGFP in *ACE2*-WT-iPSC-CMs (white arrow). (**f**) *ACE2*-WT-iPSC-CMs were incubated with SARS-CoV-2 S-RBD-sfGFP for 1 h before immunostaining on day 28 after differentiation with the indicated antibodies. The areas enclosed within the white squares are enlarged on the right side. (**g**) *ACE2*-WT-iPSC-CMs were incubated with SARS-CoV-2 S-RBD-sfGFP for 24 h before live-cell imaging with lysosomal staining on day 28 after differentiation.

We generated a recombinant S-RBD protein fused to the superfolder GFP (S-RBD-sfGFP)^35^ for live cell imaging (Supplementary Fig. S2a, b). S-RBD-sfGFP was localized to the cell membrane, perinuclear region, and partly in the cytosol of *ACE2*-WT-iPSC-CMs (Fig. 2a). The incorporated S-RBD-sfGFP was significantly decreased in *ACE2*-KO-iPSC-CMs compared to that in *ACE2*-WT-iPSC-CMs after treatment with S-RBD-sfGFP for 48 h (Fig. 2b, c, Supplementary Fig. S2c, d). To trace the initial internalization process, the iPSC-CMs were sequentially observed for 120 min after S-RBD-sfGFP treatment. Time-lapse imaging demonstrated that S-RBD-sfGFP-positive iPSC-CMs were detected approximately 10 min after S-RBD-sfGFP treatment, and these signals disappeared 48 h after the removal of S-RBD-sfGFP in *ACE2*-WT-iPSC-CMs (Fig. 2d, e, and Supplementary Figure S2e, f). To determine the endocytic pathway involved in the internalization of S-RBD-sfGFP, the iPSC-CMs were incubated with S-RBD-sfGFP for 1 h, fixed, and immunostained (Fig. 2f). S-RBD-sfGFP accumulated at the cell periphery and was dot-distributed in the cytosol of iPSC-CMs co-localized with the clathrin heavy chain, which is the major protein component of the cytoplasmic face of intracellular organelles, called coated vesicles and pits^36,37^, and EEA1 and RAB5A, which are early endosome marker proteins^38^. S-RBD-sfGFP signals did not merge with RAB7, a late endosome marker^39^, RAB11, a recycling endosome marker^40^ or caveolin-1, a scaffolding protein within the caveolar membranes^41^ in the iPSC-CMs. Twenty-four hours after treatment, S-RBD-sfGFP was co-localized with lysosomes in the iPSC-CMs (Fig. 2g). Dynamin inhibitor treatment which blocks the pathway of endocytosis^37^ significantly decreased the incorporation of S-RBD-sfGFP in the iPSC-CMs (Supplementary Figure S2g, h). These data suggested that S-RBD-sfGFP internalized into the iPSC-CMs co-localized with early endosome marker proteins and was then delivered from endosomes to lysosomes through the endolysosomal pathway in the iPSC-CMs.

### Live cell imaging demonstrates the endocytosis of SARS-CoV-2 S-RBD into the iPSC-CMs

**Figure 3:**
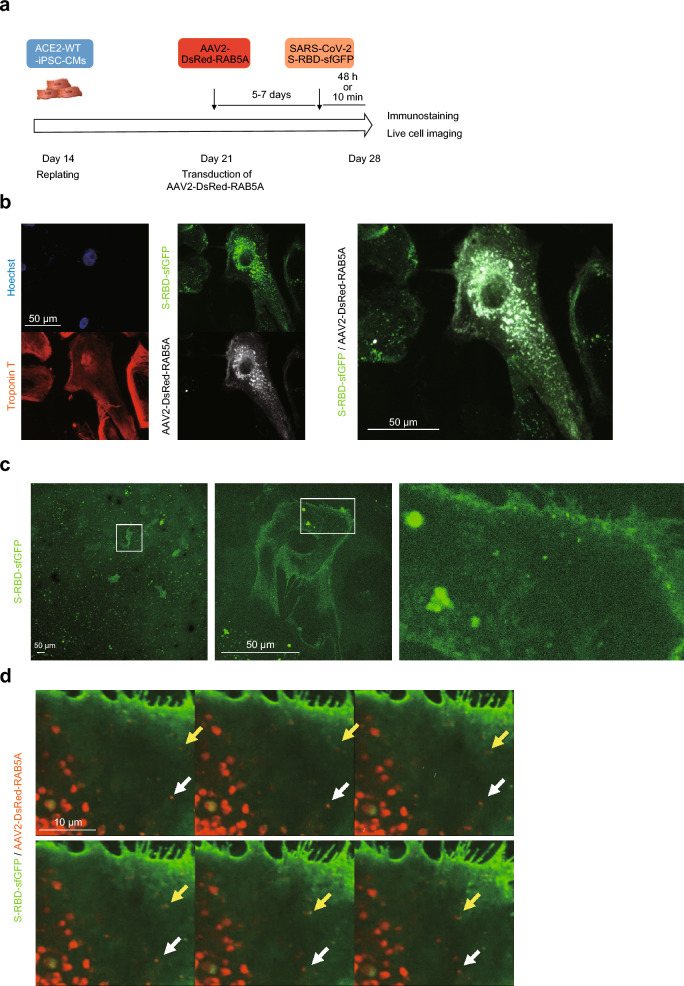
Live cell imaging of the endocytosis of SARS-CoV-2 S-RBD-sfGFP in the iPSC-CMs. (**a**) Time course of immunostaining and live cell imaging after replating the iPSC-CMs. Differentiated cardiomyocytes were replated on day 14 after differentiation and transduced with AAV2-DsRed-RAB5A at 1.0–2.0 × 10^4^ vg/cell on day 21, then incubated with SARS-CoV-2 S-RBD-sfGFP for 48 h before immunostaining or 10 min before live cell imaging on day 28 after differentiation. (**b**) *ACE2*-WT-iPSC-CMs transduced with AAV2 encoding DsRed-RAB5A were incubated with SARS-CoV-2 S-RBD-sfGFP for 48 h before immunostaining on day 28 after differentiation with the indicated antibodies. (**c**) *ACE2*-WT-iPSC-CMs were incubated with SARS-CoV-2 S-RBD-sfGFP for 10 min before live-cell imaging on day 28 after differentiation and observed by confocal microscopy. The areas enclosed within the white squares are enlarged in the right panel. (**d**) *ACE2*-WT-iPSC-CMs transduced with AAV2 encoding DsRed-RAB5A were incubated with SARS-CoV-2 S-RBD-sfGFP for 10 min before live-cell imaging on day 28 after differentiation and observed by confocal microscopy. Each dot indicated by a white or yellow arrow shows the same signal of S-RBD-sfGFP co-localized with AAV2-DsRed-RAB5A.

To further visualize the endocytosis of S-RBD into the iPSC-CMs using live-cell imaging, we generated an adeno-associated virus (AAV), encoding an N-terminal DsRed-fused full-length human *RAB5A* sequence driven by the CMV promoter (AAV2-DsRed-RAB5A). We selected the AAV2 serotype, as AAV2 has been shown to efficiently transduce the iPSC-CMs^42,43^. *ACE2*-WT-iPSC-CMs were transduced with AAV2-DsRed-RAB5A, incubated for 5–7 days, and treated with S-RBD-sfGFP (Fig. 3a). Immunostaining confirmed that S-RBD-sfGFP signals co-localized with DsRed-RAB5A in *ACE2-*WT-iPSC-CMs (Fig. 3b). Sequential observation of S-RBD-sfGFP-positive cardiomyocytes using confocal microscopy demonstrated that S-RBD-sfGFP was bound to the cell membrane, co-localized with DsRed-RAB5A, and delivered to the cytosol (Fig. 3c, d and Supplementary Video). Live cell imaging demonstrated the endocytosis of S-RBD bound to RAB5A in the iPSC-CMs.

### SARS-CoV-2 S-RBD significantly upregulate pro-inflammatory cytokines associated with innate immune response in the differentiated iPSC-CMs

**Figure 4:**
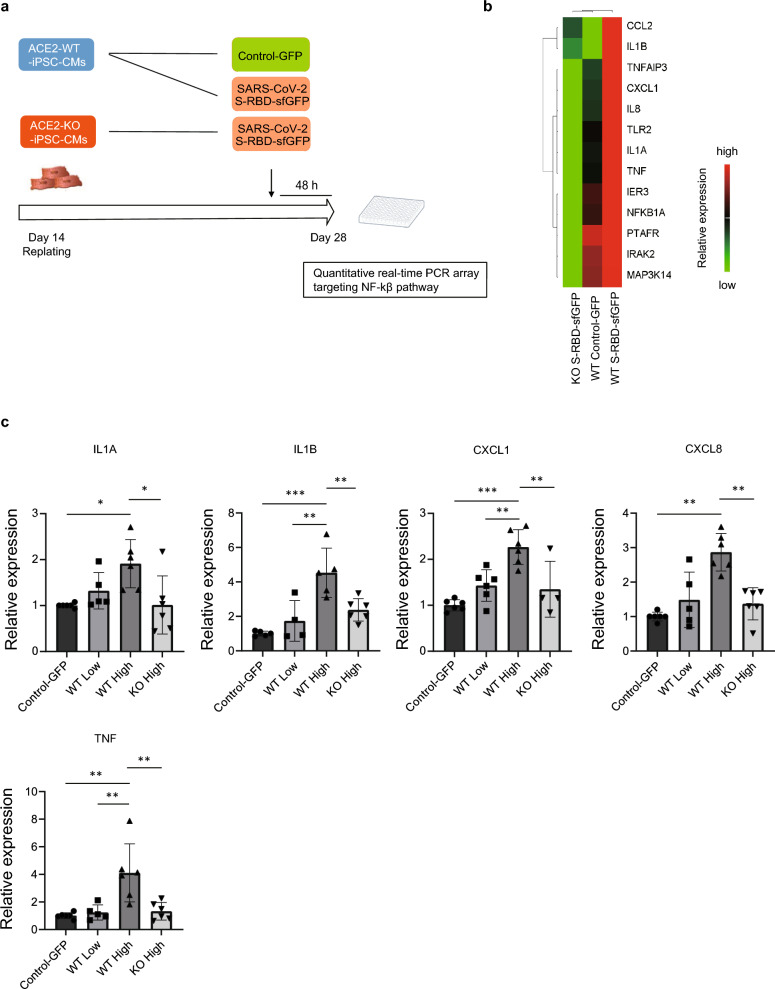
Upregulation of pro-inflammatory cytokines after S-RBD treatment depending on ACE2. (**a**) Time course of quantitative real-time PCR array analysis after replating the iPSC-CMs. Differentiated cardiomyocytes were replated on day 14 after differentiation and incubated with 1,800 ng/mL S-RBD-sfGFP and control-GFP for 48 h before quantitative real-time PCR array analysis on day 28 after differentiation. (**b**) Heat map of the selected differentially expressed genes that were upregulated in *ACE2*-WT-iPSC-CMs treated with S-RBD-sfGFP compared to control- and *ACE2*-KO-iPSC-CMs treated with S-RBD-sfGFP. The color scale denotes relative gene expression across groups (high red and low green). (**c**) The mRNA expression levels of *IL1A*, *IL1B*, *CXCL1*, *CXCL8,* and *TNF* were normalized by *GAPDH* expression and validated using quantitative real-time PCR. *ACE2*-WT-iPSC-CMs were treated with S-RBD-sfGFP for 48 h at low (600 ng/mL) and high (1,800 ng/mL) doses. *ACE2*-KO-iPSC-CMs were then treated with high doses of S-RBD-sfGFP. Relative expression levels were normalized to the expression levels in each iPSC-CM treated with control-GFP (n = 4–6 independent biological replicates). Data are presented as the mean ± SD. Statistical differences were calculated using one-way ANOVA followed by the Tukey–Kramer test for multiple comparisons. *p < 0.05, **p < 0.01, ***p < 0.001.

S-RBD protein causes inflammation and injury and activates NF-kβ pathway in mouse lung tissues^30^. To assess the effect of S-RBD on the innate immune response, we firstly screened 92 NF-kβ pathway associated genes after S-RBD treatment by quantitative real-time PCR array analysis in the iPSC-CMs including approximately 90% of troponin T-positive cardiomyocytes under our experimental conditions^32^. *ACE2*-WT-iPSC-CMs were treated with S-RBD-sfGFP or control GFP for 48 h. *ACE2*-KO-iPSC-CMs treated with S-RBD-sfGFP served as an additional control (Fig. 4a). Thirteen genes were detected to be upregulated in *ACE2*-WT-iPSC-CMs treated with S-RBD-sfGFP compared to control-GFP and *ACE2*-KO-iPSC-CMs treated with S-RBD-sfGFP using a quantitative real-time PCR array targeting the NF-kβ pathway (Fig. 4b). Quantitative real-time PCR analysis confirmed that among these genes, *IL1A, IL1B*, *CXCL1*, *CXCL8, and TNF,* which were pro-inflammatory cytokines activated by the NF-kβ signaling pathways ^44^*,* were significantly upregulated in *ACE2*-WT-iPSC-CMs in a dose-dependent manner, but not in *ACE2*-KO-iPSC-CMs (Fig. 4c). These results suggest that pro-inflammatory cytokines are upregulated after S-RBD treatment, depending on ACE2.

### Single cell RNA-sequencing reveals upregulation of IFN-responsive genes in the mature iPSC-CMs after SARS-CoV-2 S-RBD treatment

**Figure 5:**
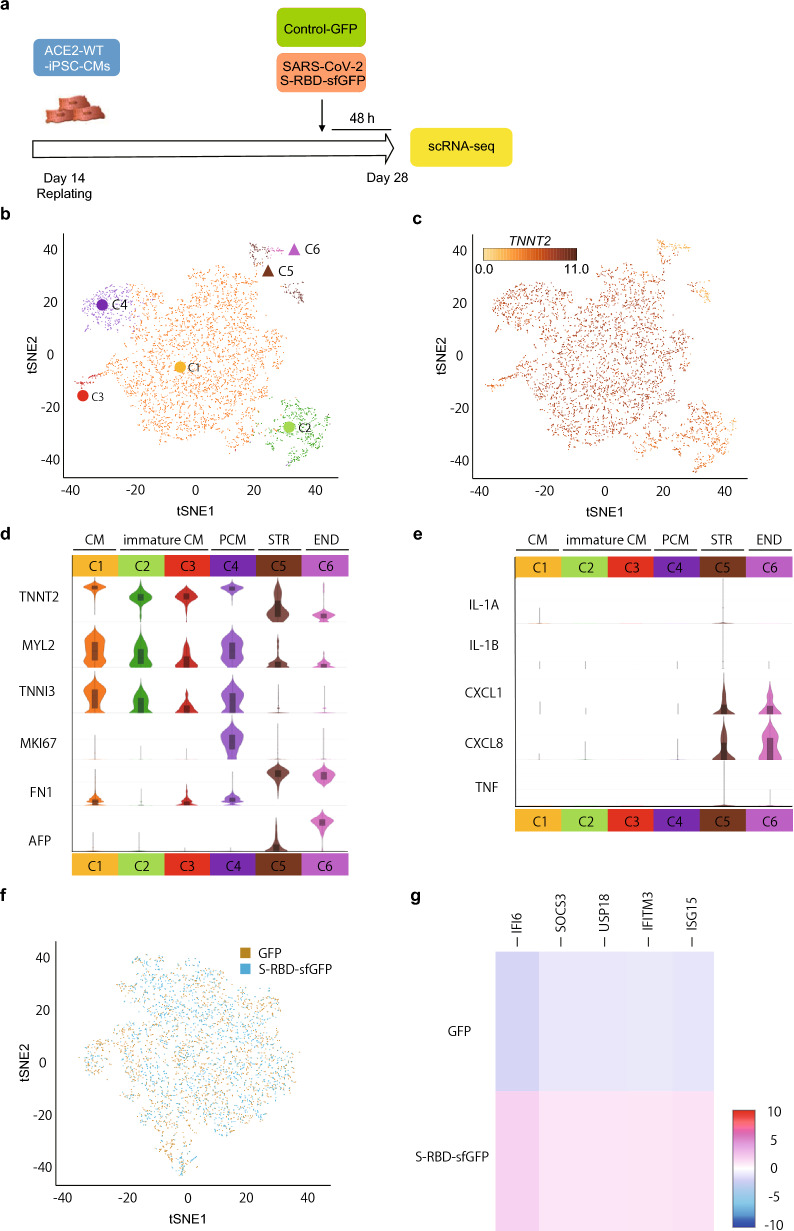
Transcriptional profiling of the iPSC-CMs subpopulations by scRNA-seq. (**a**) Time course of scRNA-seq after replating the iPSC-CMs. Differentiated cardiomyocytes were re-plated on day 14, after differentiation and incubated with 1800 ng/mL S-RBD-sfGFP and control-GFP for 48 h before scRNA-seq on day 28, after differentiation. (**b**) The iPSC-CMs treated with S-RBD-sfGFP and control-GFP (S-RBD-sfGFP = 2315 cells, GFP = 2731 cells; n = 5,046 total cells) were clustered using the k-means method (six clusters are indicated by color) and visualized using t-SNE. Circles indicate cardiomyocyte clusters (*TNNT2* positive; C1, C2, C3, and C4), including the proliferative cardiomyocyte cluster (*TNNT2* positive /*MKI67* positive; C4), and triangles indicate differentiated non-cardiomyocyte cell clusters (i.e., all other *TNNT2* negative clusters). (**c**) The same t-SNE as B is colored according to the transcript abundance of the cardiomyocyte marker cardiac troponin T (*TNNT2*). (**d**) Violin plots show the normalized transcript abundance of cell-type marker genes in each cluster. Boxplots are presented as medians and interquartile ranges, and whiskers represent the 5^th^ and 95^th^ percentiles. CM = cardiomyocyte, PCM = proliferative CM, STR = stromal-like, END = endodermal. (**e**) Violin plots showing normalized transcript abundance of genes associated with pro-inflammatory cytokines in each cluster. Boxplots are presented as medians and interquartile ranges, and whiskers represent the 5th and 95th percentiles. CM = cardiomyocyte, PCM = proliferative CM, STR = stromal-like, END = endodermal. (**f**) Mature cardiomyocytes treated with S-RBD-sfGFP and control-GFP (S-RBD-sfGFP = 1720 cells, GFP = 2,020 cells; n = 3742 total cells) were clustered by sample and visualized using t-SNE. (**g**) Differentially expressed genes were identified between the pairs of clusters corresponding to mature cardiomyocytes treated with S-RBD-sfGFP or control GFP. Heatmap showing the top five upregulated genes in the mature cardiomyocytes treated with S-RBD-sfGFP from pairwise cluster comparison. Grid cells are colored by a gene’s Log2 fold change in its cluster row (high red and low blue).

The iPSC-CMs are heterogeneous and consist of various cell populations, including mature cardiomyocytes, immature cardiomyocytes, and non-cardiomyocytes, showing distinct transcriptional states as revealed by scRNA-seq analysis^45–47^. To determine the transcriptional response in mature cardiomyocytes under the experimental conditions in which S-RBD treatment upregulated pro-inflammatory cytokines, we performed scRNA-seq on cell populations of *ACE2*-WT-iPSC-CMs treated with S-RBD-sfGFP or control GFP for 48 h (Fig. 5a). The tSNE plot obtained from 5,046 cells (n = 2315 cells [S-RBD-sfGFP] and n = 2731 cells [control GFP]) identified six clusters representing two major categories of cells: cardiomyocytes and differentiated non-cardiomyocytes (Fig. 5b). Four of the six clusters (C1, C2, C3, and C4) contained cardiomyocytes based on the expression of *TNNT2*, a conventional marker of differentiated cardiomyocytes, comprising the majority (more than 90%) of the differentiated cell populations (Fig. 5c, d). Non-cardiomyocytes contained two clusters: *FN1*-positive stromal cells (C5) and endodermal cells expressing both *FN1* and *AFP* (Fig. 5d), as previously described using scRNA-seq analysis^45^. Although quantitative real-time PCR analysis detected upregulation of pro-inflammatory cytokines, the expression levels of inflammatory genes (*IL1A*, *IL1B*, and *TNF*) detected by scRNA-seq exhibited low average counts (less than one count per cell across the entire dataset). *CXCL1* and *CXCL8* were expressed predominantly in the non-cardiomyocyte clusters, but not in the cardiomyocytes (Fig. 5e), and *CXCL1* was significantly upregulated in these non-cardiomyocyte clusters after S-RBD-sfGFP treatment (Supplementary Fig. S3a).

*MYL2* and *TNNI3*, which are marker genes of cardiomyocyte maturation^48^, were increased in clusters C1 and C4 (Fig. 5d), and *MKI67*, a marker of cell proliferation, was increased in cluster C4, suggesting that cluster C1 represents mature cardiomyocytes. To reveal the transcriptional response in mature cardiomyocytes, we analyzed the differential expression in the reclustered C1 subset (n = 1720 cells [S-RBD-sfGFP], n = 2022 cells [control GFP], Fig. 5f) and found that the IFN-responsive genes (*IFI6*, *ISG15*, *IFITM3*, *SOCS3,* and *USP18*) were significantly upregulated in mature cardiomyocytes treated with S-RBD-sfGFP compared to those treated with control GFP (Fig. 5g and Supplementary Fig. S3b).

### SARS-CoV-2 S-RBD promotes protein ISGylation in the iPSC-CMs

**Figure 6:**
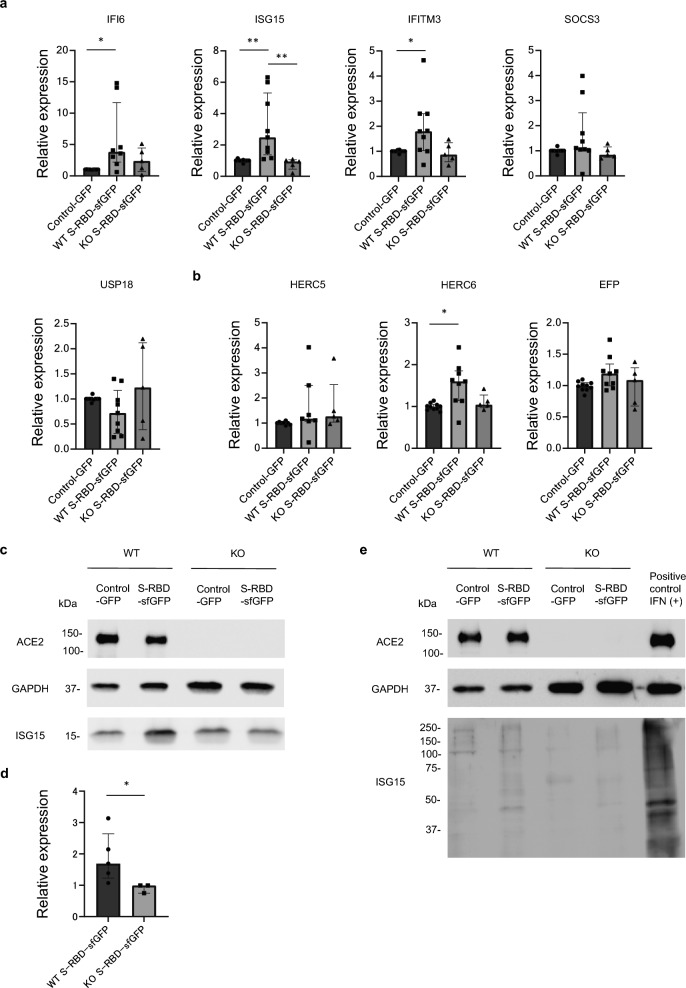
Protein ISGylation in the iPSC-CMs after S-RBD treatment. (**a**) The mRNA expression levels of *IFI6, ISG15, IFITM3, SOCS3,* and *USP18* were normalized by *GAPDH* expression and validated using quantitative real-time PCR. *ACE2*-WT and *ACE2*-KO iPSC-CMs were treated with 1800 ng/mL S-RBD-sfGFP for 48 h. Relative expression levels were normalized to the expression levels in each iPSC-CM treated with control GFP (n = 5–10 independent biological replicates). Data are presented as medians and interquartile ranges. Statistical differences were calculated using the Kruskal–Wallis test followed by Dunn’s test for multiple comparisons. *p < 0.05, **p < 0.01. (**b**) The mRNA expression levels of *HERC5, HERC6,* and *EFP* were normalized by *GAPDH* expression and validated using quantitative real-time PCR. *ACE2*-WT and *ACE2*-KO iPSC-CMs were treated with 1800 ng/mL S-RBD-sfGFP for 48 h. Relative expression levels were normalized to the expression levels in each iPSC-CM treated with control GFP (n = 5–10 independent biological replicates). Data are presented as medians and interquartile ranges. Statistical differences were calculated using the Kruskal–Wallis test followed by Dunn’s test for multiple comparisons. *p < 0.05. (**c**) Whole cell lysates were extracted from *ACE2*-WT-iPSC-CMs and *ACE2*-KO-iPSC-CMs on day 28 after differentiation and analyzed by western blotting using the indicated antibodies. Both iPSC-CMs were incubated with 1200 ng/mL SARS-CoV-2 S-RBD-sfGFP or control GFP for 48 h prior to western blotting. Original blots are presented in Supplementary Fig. S6. (**d**) Quantified ISG15 protein expression levels were normalized by GAPDH expression in *ACE2*-WT-iPSC-CMs and *ACE2*-KO-iPSC-CMs treated with S-RBD-sfGFP (n = 3–5). Data are presented as medians and interquartile ranges. Statistical differences were calculated using the Mann–Whitney U test. *p < 0.05. (**e**) Whole cell lysates were extracted from *ACE2*-WT-iPSC-CMs and *ACE2*-KO-iPSC-CMs on day 28 after differentiation and analyzed by western blotting using the indicated antibodies. Both iPSC-CMs were incubated with 6000 ng/mL SARS-CoV-2 S-RBD-sfGFP, control-GFP, or 10 IU/mL IFN as a positive control for 48 h before western blotting. Original blots are presented in Supplementary Fig. S6.

Quantitative real-time PCR analysis confirmed that among the five genes upregulated in mature cardiomyocytes, *IFI6*, *ISG15*, and *IFITM3* were significantly upregulated in *ACE2*-WT-iPSC-CMs but not in *ACE2*-KO-iPSC-CMs after S-RBD-sfGFP treatment (Fig. 6a). We further promoted the maturation of the iPSC-CMs using electrical stimulation and confirmed that *IFI6*, *ISG15*, and *IFITM3* were significantly upregulated in these iPSC-CMs (Supplementary Fig. S4a). ISG15 is a ubiquitin-like protein upregulated at both the mRNA and protein levels in patients with acute myocarditis and inflammatory cardiomyopathy^49^, and is conjugated to a variety of proteins (called ISGylation) by specific E1-E2-E3 ubiquitin cascade enzymes when cells are treated with type I IFN^50^. Quantitative real-time PCR analysis demonstrated that among the E3 ligase genes (*HERC5*, *HERC6,* and *EFP*), the expression of *HERC6* was significantly upregulated in *ACE2*-WT-iPSC-CMs after S-RBD-sfGFP treatment compared to that in the control GFP, but not in *ACE2*-KO-iPSC-CMs (Fig. 6b and Supplementary Fig. S4b). To reveal the direct effect of S-RBD treatment on ISGylation in the iPSC-CMs, protein samples extracted from the iPSC-CMs were analyzed by western blot using an anti-ISG15 antibody. S-RBD treatment significantly increased the expression levels of ISG15 in *ACE2*-WT-iPSC-CMs compared to *ACE2*-KO-iPSC-CMs (Fig. 6c, d). Western blotting analysis showed that ISGylation increased in a dose-dependent manner in *ACE2*-WT-iPSC-CMs after treatment with S-RBD-sfGFP (Supplementary Fig. S4c). Notably, the protein ISGylation observed in *ACE2*-WT-iPSC-CMs was suppressed in *ACE2*-KO-iPSC-CMs (Fig. 6e).

## Discussion

**Figure 7:**
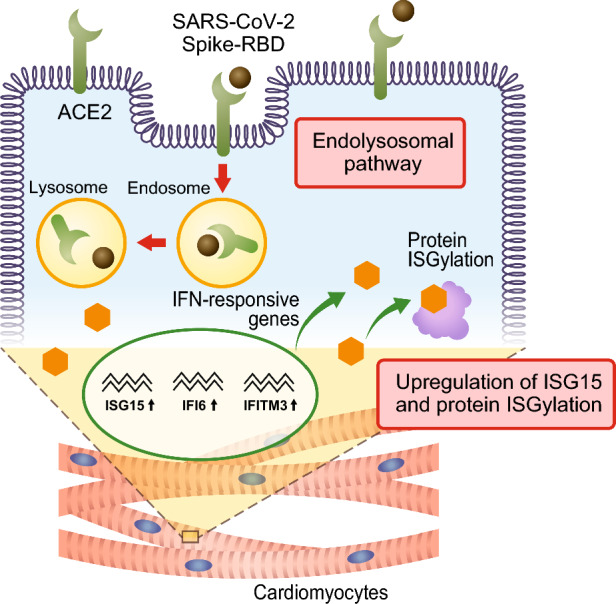
Graphical summary of the main results. S-RBD was internalized into the human iPSC-CMs via ACE2 by the endolysosomal pathway. The internalized S-RBD induced an innate immune response, upregulated IFN-responsive genes (*IFI6*, *ISG15*, and *IFITM3*) in mature cardiomyocytes, and promoted protein ISGylation in the iPSC-CMs.

In this study, we investigated the direct effects of SARS-CoV-2 S-RBD using the human isogenic iPSC-CMs with or without ACE2 expression. The main findings of this study are as follows. First, we demonstrated that S-RBD was internalized into the human iPSC-CMs via ACE2 by the endolysosomal pathway. Second, the internalized S-RBD induced an innate immune response, upregulated IFN-responsive genes in mature cardiomyocytes, and promoted protein ISGylation in the iPSC-CMs (Fig. 7). Myocarditis has been recognized as a complication of COVID-19 vaccinations, especially in adolescents and young adults^51^. Several histopathological analyses of endomyocardial biopsies or autopsies from patients with myocarditis following COVID-19 vaccination have revealed spike proteins and S-RBD in the cardiomyocytes, infiltrating predominantly CD4^+^ T lymphocytes and macrophages^19,24,25^. One of the possible explanations is direct immune activation by spike protein in cardiomyocytes but the role of the spike protein in the development of myocarditis is still unknown. It has been revealed that mRNA vaccines can result in spike protein expression in the muscle tissue, as well as in the lymphatic system and other cells including cardiomyocytes after entry into the circulation^20^. S-RBD is a key domain of spike protein which binds to ACE2 and has been implicated in inflammation^30,31^. In the human heart tissue, ACE2 is expressed in the cardiomyocytes and pericytes and human iPSC-CMs express ACE2^33^, suggesting that the human isogenic iPSC-CMs are a useful experimental tool. These facts prompted us to examine the direct effects of SARS-CoV-2 S-RBD proteins on the cardiomyocytes using human iPSC-CMs models.

In the current study, immunostaining combined with live cell imaging demonstrated that the S-RBD-sfGFP was internalized approximately 10 min after the initial treatment, co-localized with clathrin heavy chain, EEA1, and RAB5A, which are early endosome marker proteins, and then delivered to lysosomes. Immunostaining experiments using HEK293T cells treated with SARS-CoV-2 spike protein demonstrated that the spike protein undergoes rapid clathrin-mediated endocytosis, and chemical inhibition of clathrin-mediated endocytosis blocks spike protein entry^37^. Immunostaining experiments using Huh7 cells revealed that S-RBD co-localized with RAB5 but not with RAB11 after 1 h of S-RBD treatment^51^. Under our experimental conditions, internalized S-RBD-sfGFP did not merge with RAB7, a late endosome marker, or RAB11, a recycling endosome marker, suggesting that S-RBD-sfGFP may be processed via specific trafficking machinery in the human iPSC-CMs, consistent with previous findings.

NF-kβ pathway is one of the critical signaling pathways for the COVID-19 infection-induced pro-inflammatory cytokine response^52–54^. The genes related with NF-kβ signaling pathway is upregulated in the SARS-CoV-2 infected human iPSC-CMs by bioinformatics analysis^55^ and S-RBD activates NF-kβ pathway followed by the vast transcriptional activation of pro-inflammatory cytokines in mouse lung tissue^30^. Based on these findings, we evaluated the direct effect of internalized S-RBD on the innate immune response in the iPSC-CMs using quantitative PCR array analysis followed by scRNA-seq analysis, because the iPSC-CMs consist of various cell populations showing distinct transcriptional states, as revealed by scRNA-seq analysis^45–47^. S-RBD-sfGFP treatment significantly upregulated *CXCL1*, encoding chemokines involved in inflammatory response, specifically in *FN1*-positive stromal cells and both *FN1* and *AFP* positive endodermal cells, which occupied a small population (5.3%) of the differentiated iPSC-CMs. Although these stromal and endodermal cells might still be differentiating and the cell lineage of these differentiated cells remains unclear, these cell populations may recapitulate immunoreactive function and express chemokines and inflammatory genes detected by quantitative real-time PCR, enabling the evaluation of minute inflammatory responses in the iPSC-CMs exposed to S-RBD-sfGFP.

S-RBD-sfGFP treatment significantly upregulated the IFN-responsive genes (*IFI6*, *ISG15*, and *IFITM3*) in mature cardiomyocytes, suggesting that S-RBD acts as a pathogen-associated molecule and promotes the expression of IFN-responsive genes. ISG15 is a ubiquitin-like protein induced by interferons or pathogens, and is conjugated to a variety of target proteins in a process called ISGylation, which plays a role in the regulation of the immune system in mammals^50^. ISG15 is constitutively expressed in the human cardiomyocytes and is upregulated in patients with acute myocarditis and inflammatory cardiomyopathy^49^. ISG15 in the cardiomyocytes contributes to the suppression of viral replication, and the absence of protein modification with ISG15 was accompanied by profound exacerbation of myocarditis in mice infected with coxsackievirus B3^49^. More recently, experimental findings have shown that ISG15 deficiency preserves cardiac function in mice under pressure overload, suggesting that protein ISGylation is an inflammation-induced post-translational modification that may contribute to heart failure development^56^. Our current findings demonstrated that S-RBD treatment dose-dependently increased ISG15 expression and promoted protein ISGylation in the human iPSC-CMs via ACE2. However, whether protein ISGylation in the cardiomyocytes is beneficial remains unknown and requires further investigation.

This study had several limitations. First, our experiments were based on the human iPSC-CMs in vitro, which may not fully reflect the effect of S-RBDs on the heart in vivo, including the inflammatory cells. Second, because the iPSC-CMs used in this study were generated from a patient with HCM the presence of HCM might also affect the inflammatory response and the innate immunity. Third, whether the upregulation of IFN-responsive genes in the cardiomyocytes is directly promoted by internalized S-RBD or indirectly affected by the surrounding non-cardiomyocyte population remains to be elucidated. Furthermore, we did not exclude the possible mechanism of direct uptake of vaccine mRNA. Kwon et al. demonstrated that extracellular vesicles (EVs) containing the viral RNA can be transmitted into iPSC-CMs and increased the expression of the pro-inflammatory genes (*IL1B*, *IL6* and *MCP1*) 24 h after EVs treatment, suggesting the different mechanism, the time points of entry, and the upregulated genes from the internalization of S-RBD protein. Circulating vaccine mRNA was detected in plasma for several weeks after vaccination^57^, and intravenous injection of vaccine mRNA may induce myocarditis in mouse model^58^. These results indicate direct vaccine mRNA delivery into cardiomyocytes. Fourth, the concentration of S-RBD used for imaging experiments and transcriptional profiling (> 600 ng/mL) was lower than the estimated serum concentration of S protein after SARS-CoV-2 infection (2500–17,500 ng/mL)^59^ but higher than the concentration after mRNA vaccination (20–100 pg/mL)^22,23^, suggesting that our experimental findings using the iPSC-CMs do not necessarily represent a physiological response in human patients. Nonetheless, because protein subunit COVID-19 vaccine using S-RBD has been developed to clinical stage^60,61^, our findings provide a basic evidence when considering the risks and benefits of vaccination.

In conclusion, SARS-CoV-2 S-RBD was internalized via ACE2 through the endolysosomal pathway, upregulated the IFN-responsive genes, and promoted ISGylation in the human iPSC-CMs. These findings may further advance our understanding of S-RBD-mediated pathophysiology in the human cardiomyocytes.

## Methods

Details of the materials and methods used in in this study are available in Supplementary Methods.

### Human samples

The use of human samples and genomic analysis was approved by the Ethics Committee of Osaka University Hospital, and written informed consent was obtained from all participants. This investigation conformed to the Ethical Guidelines for Medical and Health Research Involving Human Subjects in Japan and all principles outlined in the Declaration of Helsinki.

### Generation of isogenic ACE2-KO-iPSC-CMs using CRISPR/Cas9 genome editing

Plasmid constructs for genome editing were transfected into iPSCs as previously described^62,63^ with some modifications. We generated a pX459 vector^64^ encoding a gRNA that targeted a protospacer adjacent motif sequence in exon 2 of ACE2 and introduced non-homologous end-joining in the ACE2 gene on the X chromosome.

### S-RBD fused to superfolder GFP (S-RBD-sfGFP) production

Expi293F cells (Thermo Fisher Scientific, Waltham, Massachusetts, USA) were cultured in Expi293 Expression Medium (Thermo Fisher Scientific) at 125 rpm, 8% CO2, and 37 °C. To produce S-RBD-sfGFP or GFP as controls, the cells were prepared at a concentration of 3 × 10^6^ /mL. pcDNA3-SARS-CoV-2-S-RBD-sfGFP (Addgene plasmid # 141184)^35^ was used to generate the recombinant S-RBD-sfGFP. A control plasmid encoding GFP sequence under the control of the CMV promoter was used. These plasmids were transfected in to the Expi293F cells using Expifectamine (Thermo Fisher Scientific) according to the manufacturer's manual, with Transfection Enhancers added 18–22 h post-transfection, and medium supernatant harvested after 60 h. The cells were removed by centrifugation at 300×*g* for 10 min, the remaining cell debris and precipitates were then removed by centrifugation at 20,000×*g* for 5 min and medium supernatant was stored at − 80 °C. iPSC-CMs were incubated with 600–1200 ng/mL SARS-CoV-2 S-RBD-sfGFP before immunostaining or live cell imaging 28 days after differentiation.

### Single cell dissociation for scRNA-seq, library preparation, and sequencing

iPSC-CMs were plated at 3 × 10^5^ cells/well in 12-well plates on day 21 after differentiation and treated with S-RBD-sfGFP or control-GFP at 1800 ng/mL for 48 h on day 26–28. For single cell dissociation, the cardiomyocyte wells were washed with Dulbecco’s PBS twice and incubated with pre-warmed 0.25% Trypsin–EDTA for 8–10 min at 37 °C. Monolayers were gently dissociated using a P1000 micropipette to obtain single cells, collected, and added to 1 mL of Dulbecco’s modified Eagle’s medium (DMEM) containing 10% fetal bovine serum (FBS) and PSG and filtered with 100 μm cell strainer. The cell suspension was centrifuged at 180 g for 5 min at 4 °C. The single cell suspension was gently resuspended in 1 mL of DMEM containing 10% FBS and PSG and counted twice using a hemocytometer to obtain the total cell count for each sample. For scRNA-seq, a Chromium Next GEM Single Cell 3′ Reagent Kit (V3.1) (10 × Genomics, Pleasanton, California, USA) was used. The samples were loaded onto chips at an optimized concentration to capture approximately 5000 cells in individual 1-cell droplets. Single-cell libraries were sequenced using an Illumina HiSeq X System.

### scRNA-seq data processing, clustering, and visualization

Raw base call files for each sample were demultiplexed and converted to FASTQ files using the 10 × Genomics toolkit Cell Ranger 7.0.1 (cellranger mkfastq). All single-cell transcriptome sequencing data were aligned and quantified using Cell Ranger 7.0.1 against the GRCh38/hg38 human reference genome. Gene expression counts were performed using a cell ranger count for each sample and then aggregated with a single instance of cell ranger aggr. Unique Molecular Identifiers (UMI) were filtered and corrected to 10–17 UMIs per Barcode (Log2). We then filtered out outlier genes that were outside the range of seven genes per barcode (Log2) to remove noise due to sequencing depth or cell conditions. Furthermore, cells above 20% of percent mitochondrial UMI counts were also removed to eliminate low quality cells. Data dimensionality reduction by t-SNE and unsupervised clustering by k-means clustering were visualized using Loupe Browser v6.4.0. Cluster IDs were assigned after the clustering.

### Statistical analysis

Data are presented as medians and interquartile ranges from at least three independent experiments, unless otherwise stated. All statistical analyses were performed using the SPSS software version 25 (IBM Corp., Armonk, New York, USA). Graphs were generated using GraphPad Prism v9.1.0 (GraphPad Software, Inc., La Jolla, California, USA). The data were analyzed using the Shapiro–Wilk test to test for normal distribution and the Levene test for equal variances. For data with a normal distribution, Student’s *t-*test was used to compare two groups, and one-way ANOVA followed by the Tukey–Kramer test as a post-hoc analysis was used to compare multiple groups. If the data were not normally distributed, the Mann–Whitney U test was used to compare two groups, and the Kruskal–Wallis test followed by Dunn’s test as a post-hoc analysis was used to compare multiple groups. Statistical significance was set at *p* < 0.05.

### Supplementary Information

Supplementary Figures.

Supplementary Information.

Supplementary Tables.

Supplementary Video 1.

## Data Availability

The data that support the findings of this study are available from the corresponding author upon reasonable request. The scRNA-seq data reported in this paper have been deposited at the Genomic Expression Archive (GEA) under the accession number E-GEAD-628.
