# Supplementary material for: SARS-CoV-2 spike receptor-binding domain is internalized and promotes protein ISGylation in human induced pluripotent stem cell-derived cardiomyocytes
Source: Sci Rep. 2023 Dec 4;13:21397. doi: 10.1038/s41598-023-48084-7 (PMC10696029; doi:10.1038/s41598-023-48084-7)

Supplementary_Figure_1

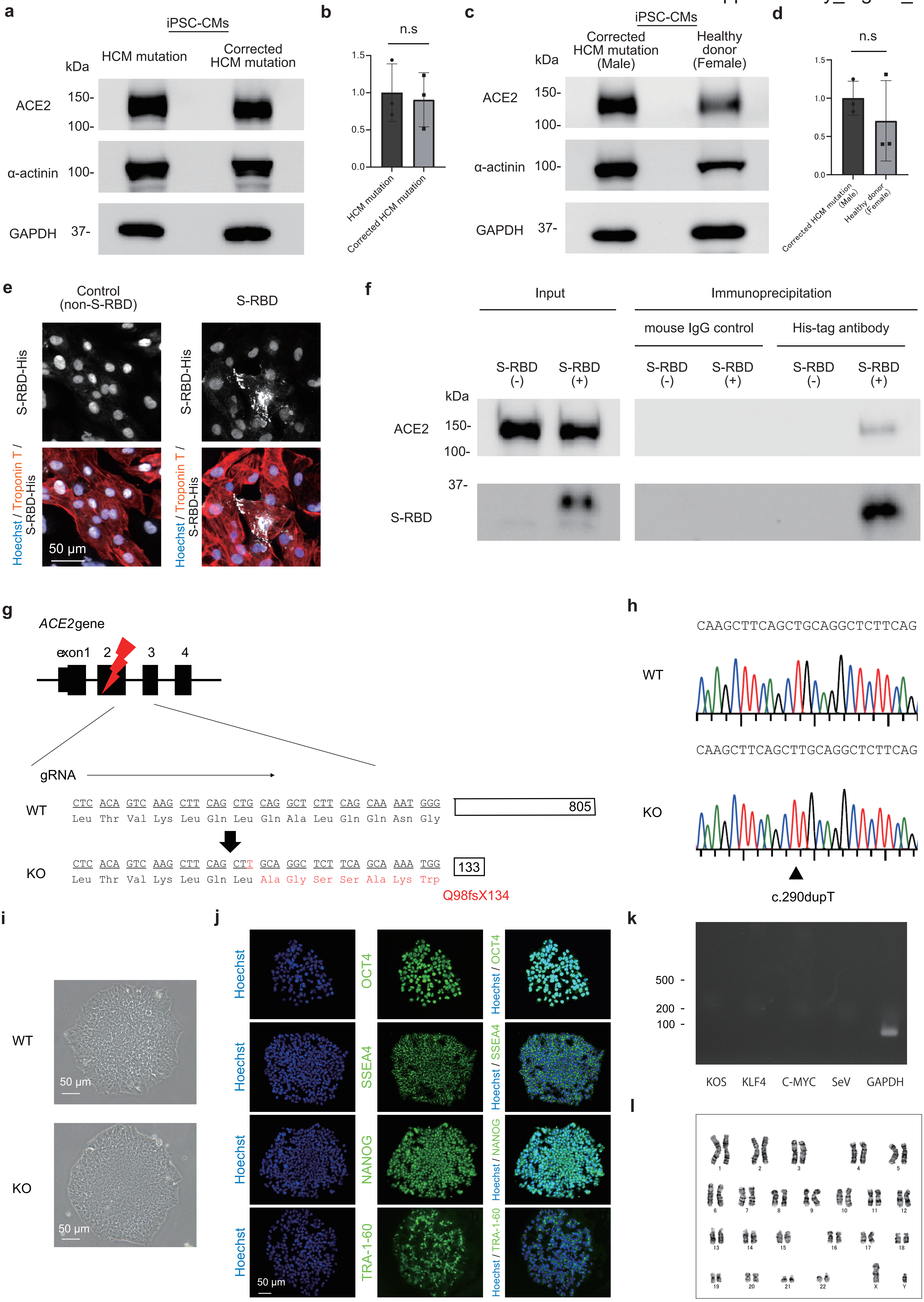

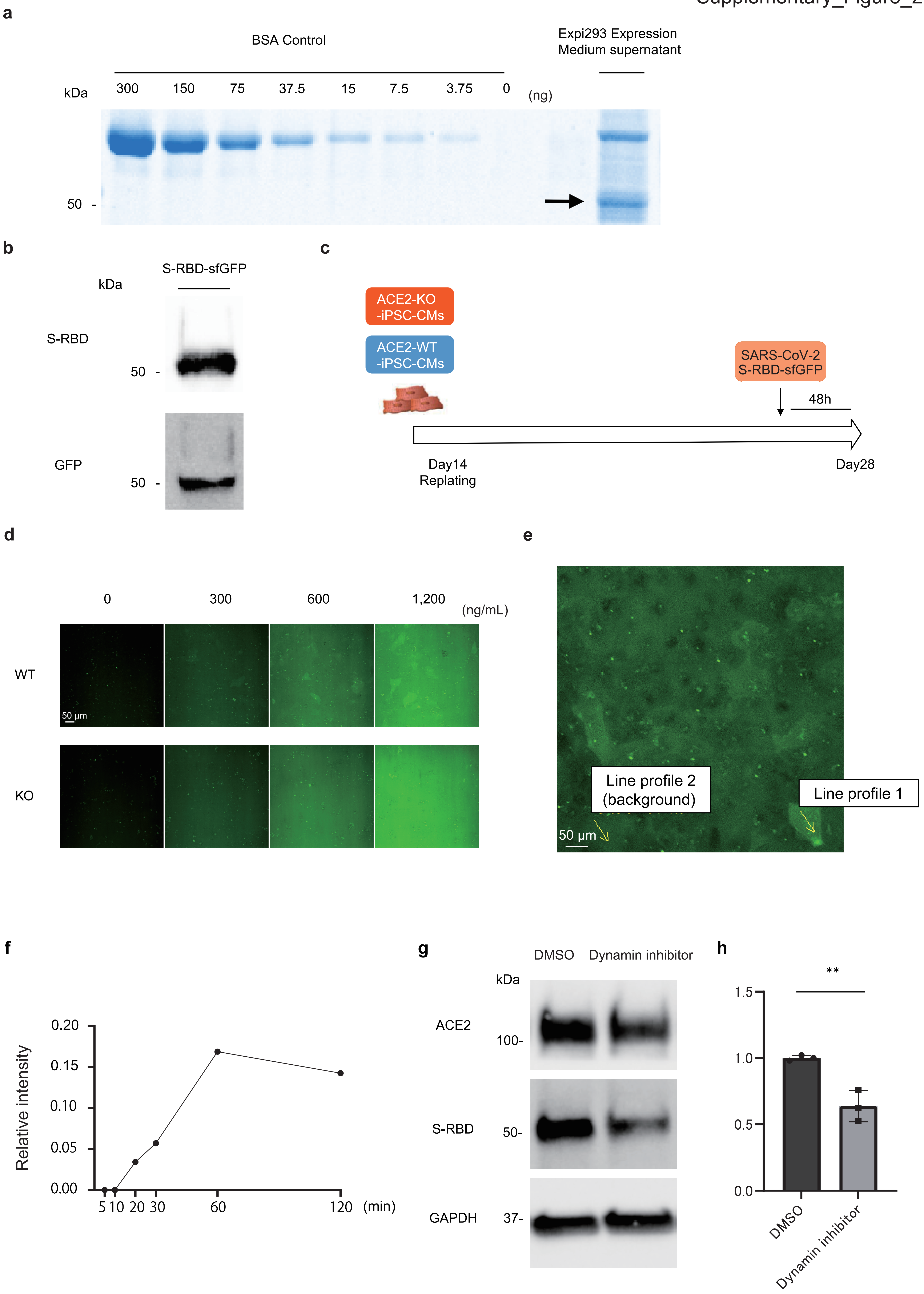
a
BSA Control
Expi293 Expression Medium supernatant
kDa
300
150
75
37.5
15
7.5
3.75
0
(ng)
50 -
b
S-RBD-sfGFP
kDa
S-RBD
50 -
GFP
50 -
c
ACE2-KO -iPSC-CMs
ACE2-WT -iPSC-CMs
SARS-CoV-2 S-RBD-sfGFP
48h
Day14 Replating
Day28
d
0
300
600
1,200
(ng/mL)
WT
KO
50 μm
e
Line profile 2 (background)
Line profile 1
50 μm
f
Relative intensity
0.20
0.15
0.10
0.05
0.00
5 10 20 30
60
120
(min)
g
DMSO
Dynamin inhibitor
kDa
ACE2
100-
S-RBD
50-
GAPDH
37-
h
**
1.5
1.0
0.5
0.0
DMSO
Dynamin inhibitor

Supplementary_Figure_3

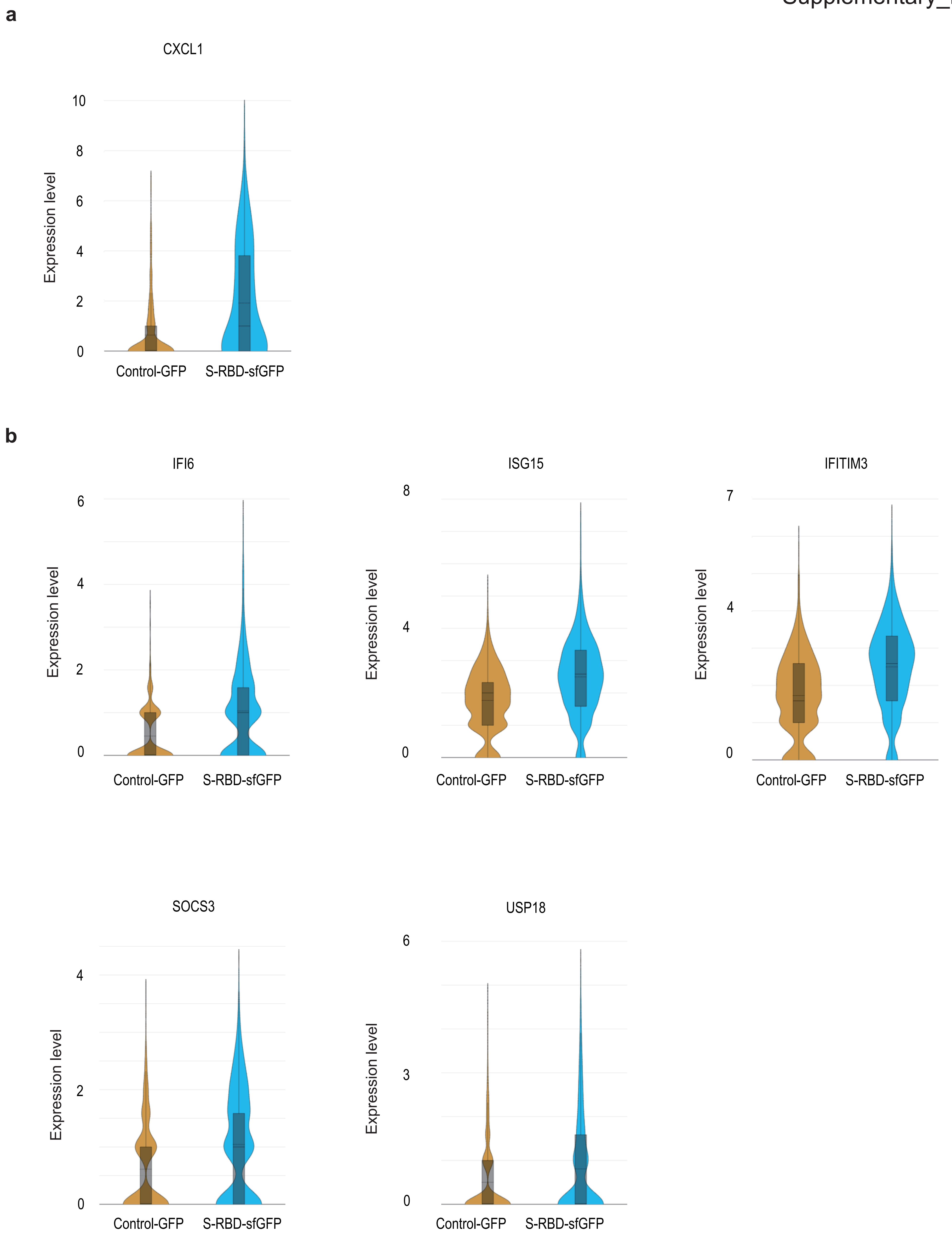

Supplementary_Figure_4

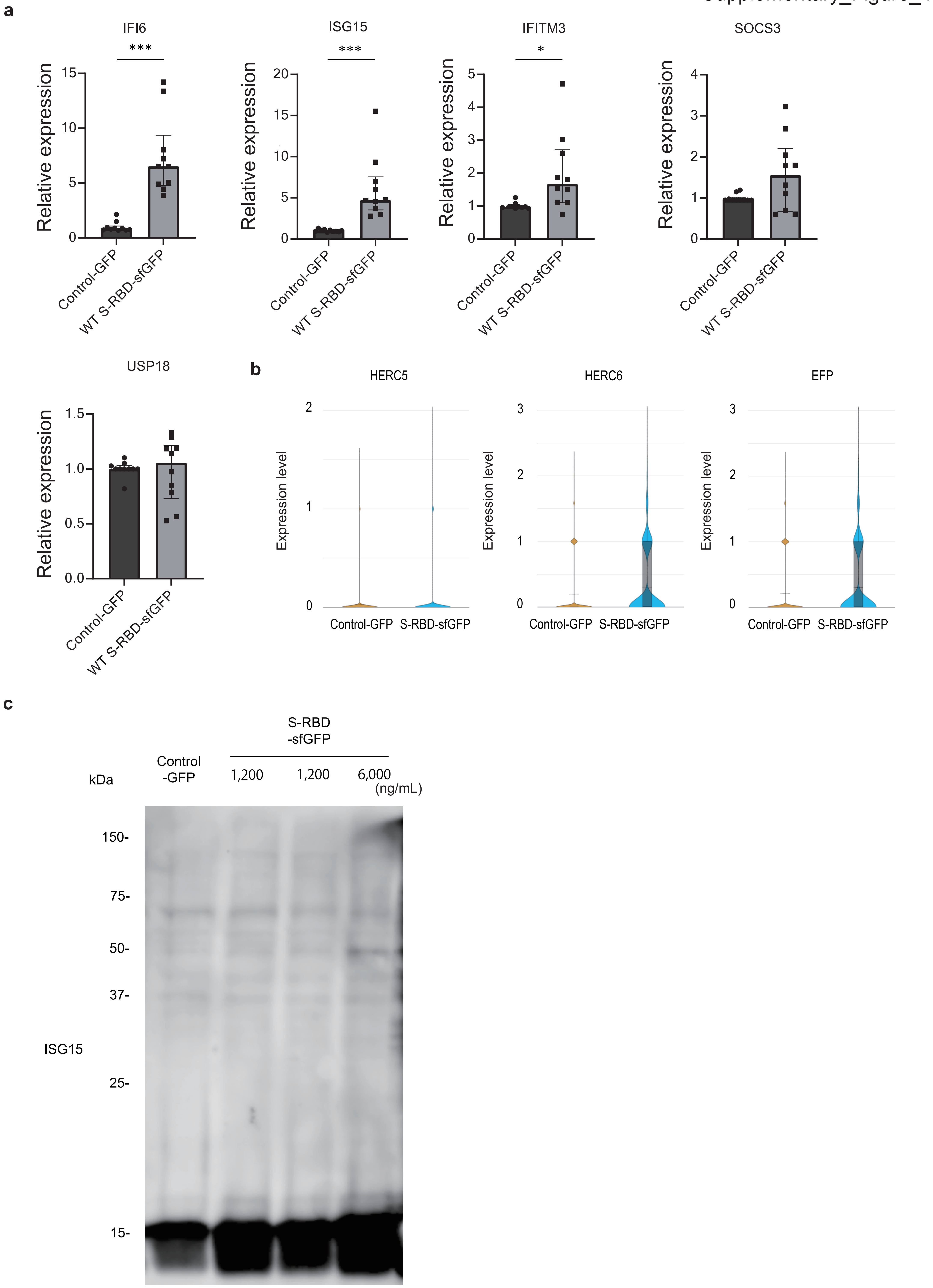

Fig. 1

ACE2

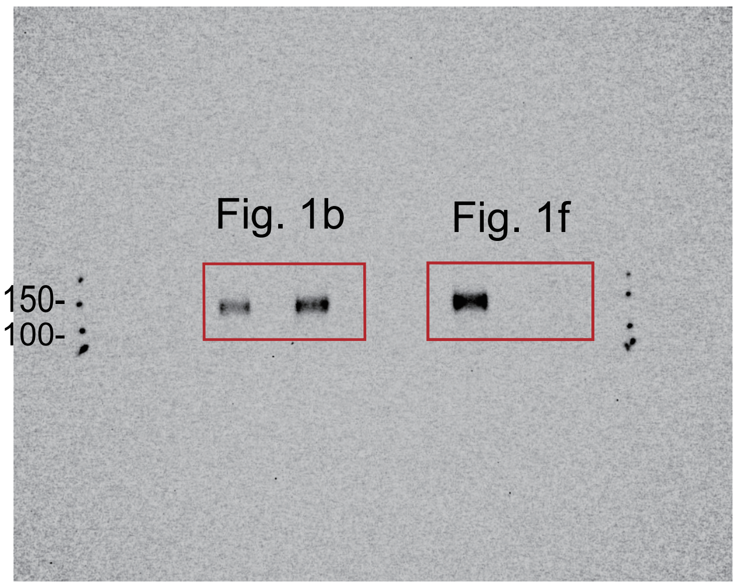

α-actinin

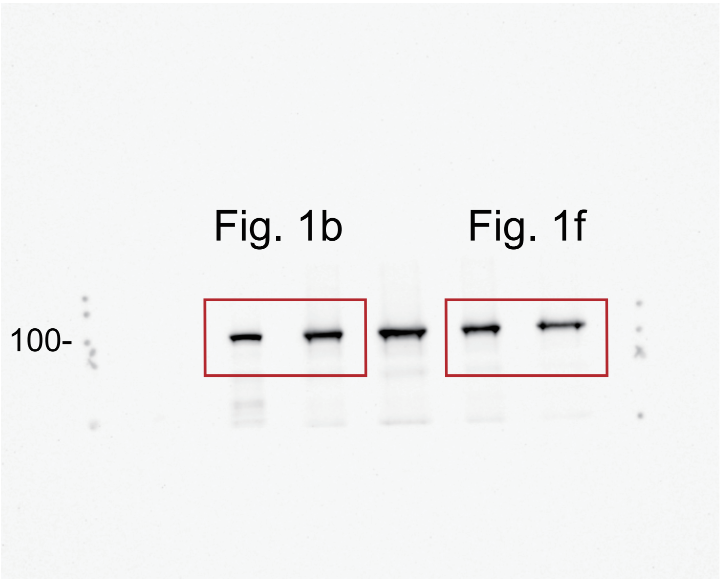

GAPDH

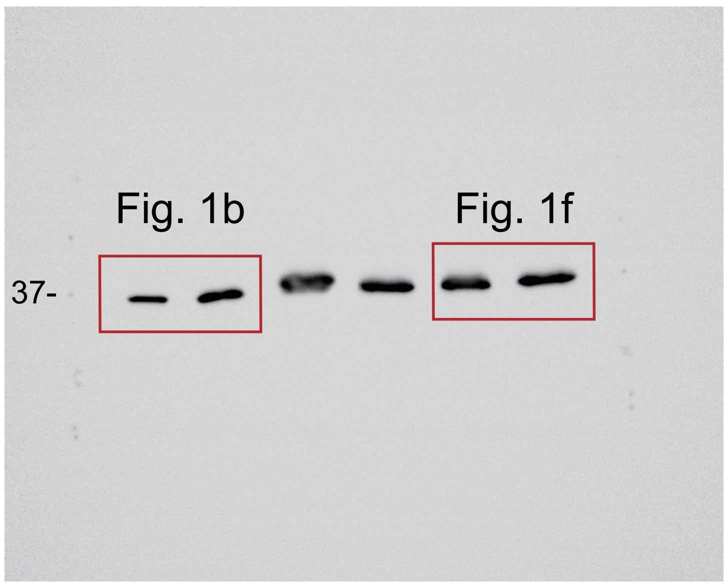

Fig. 2b

ACE2

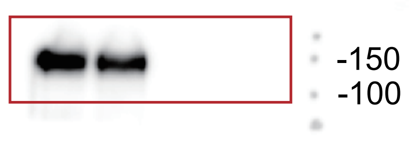

α-actinin

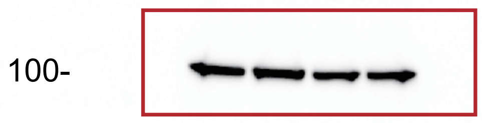

S-RBD

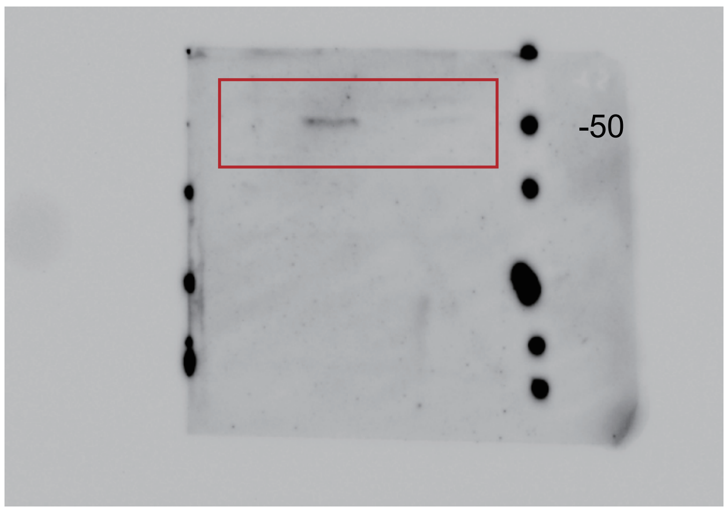

GAPDH

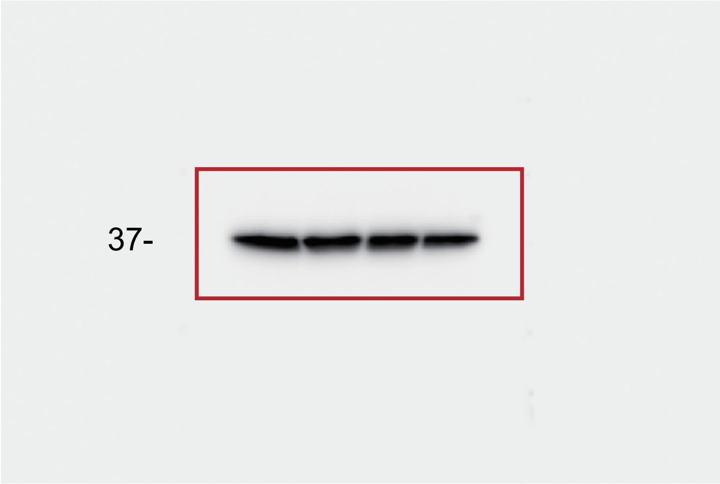

Fig. 6c

ACE2

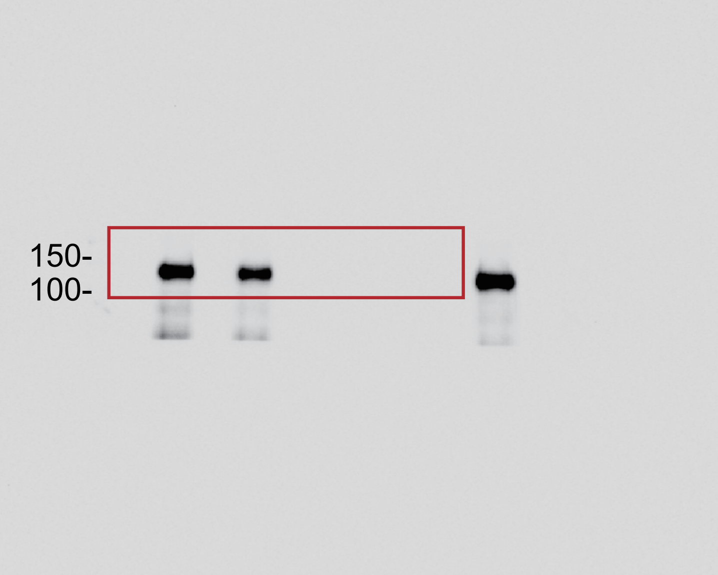

ISG15

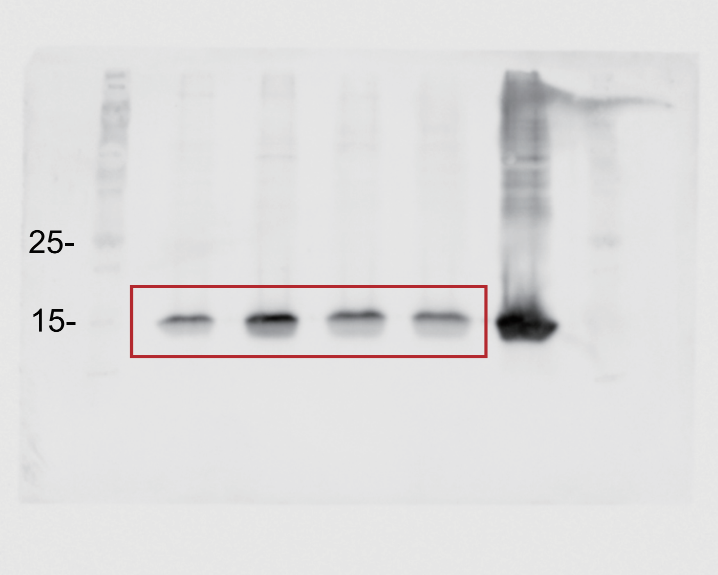

GAPDH

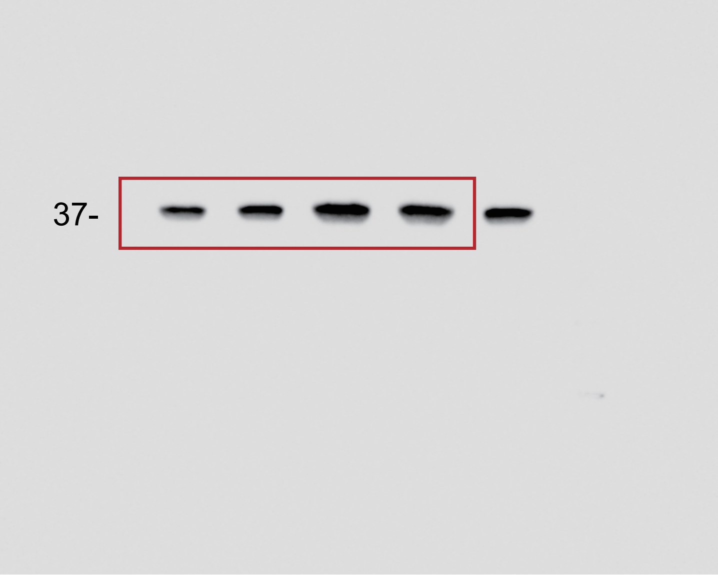

Fig. 6e

ACE2

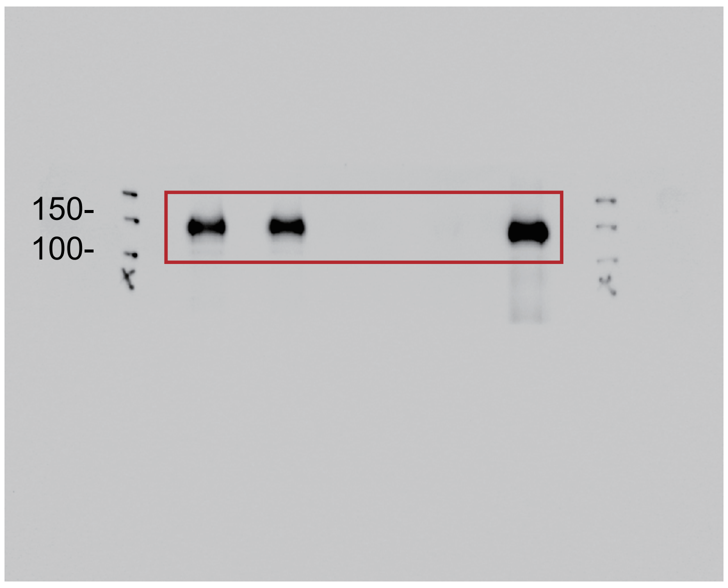

ISG15

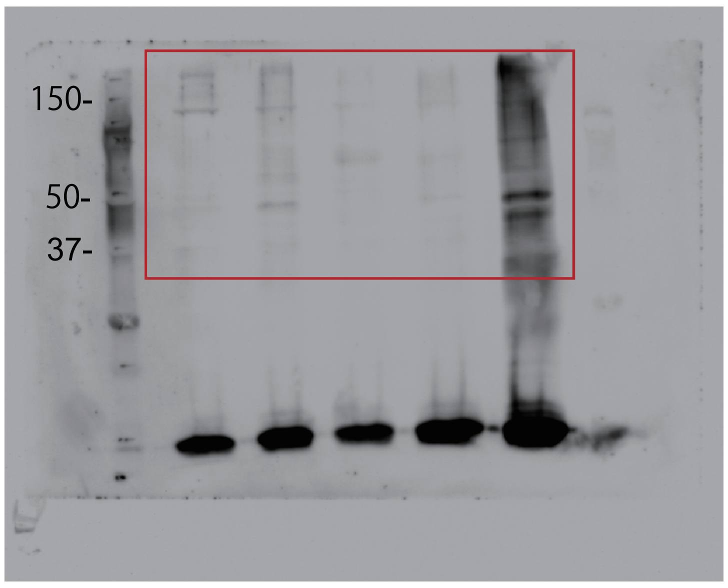

GAPDH

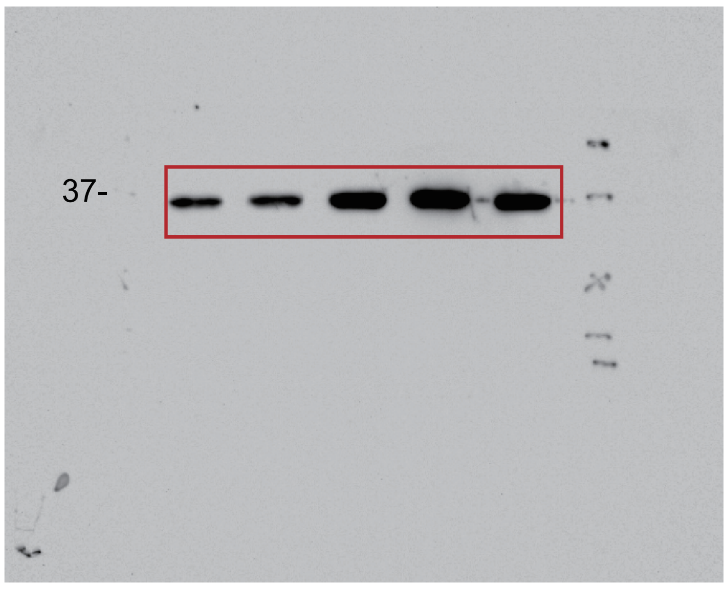

Fig. S1a

ACE2

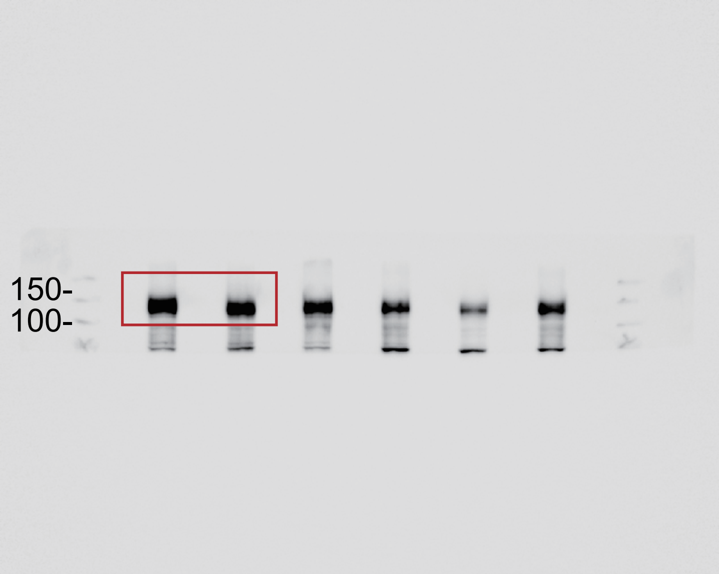

α-actinin

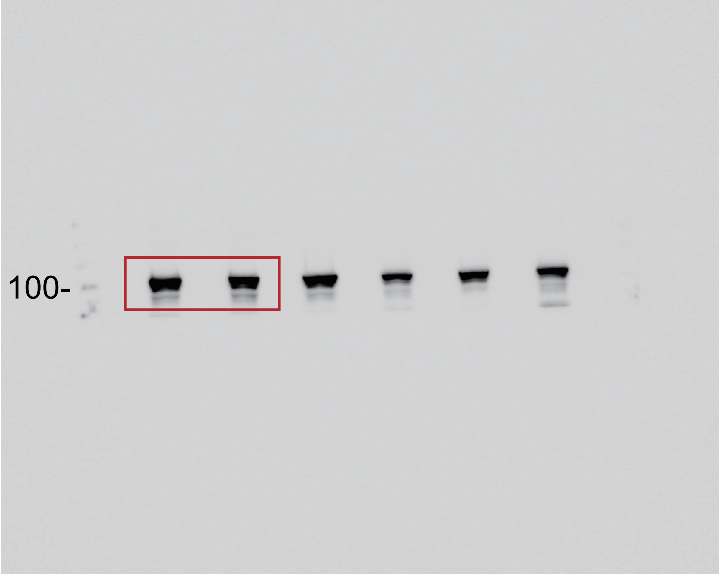

GAPDH

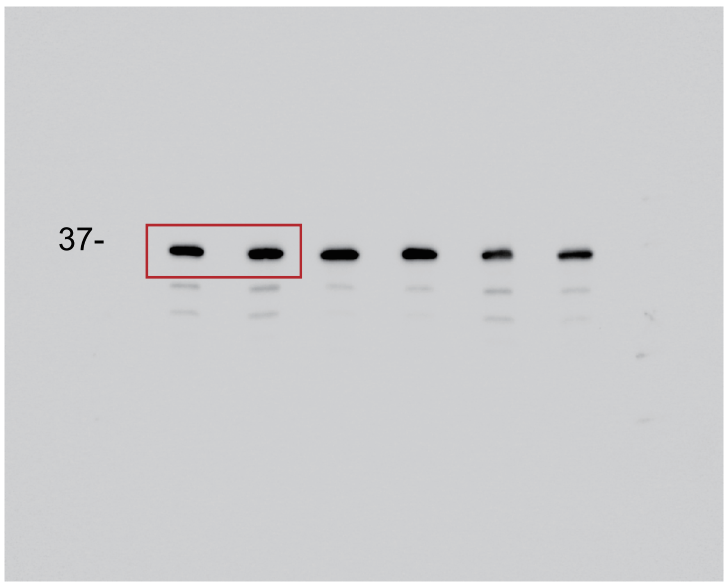

Fig. S1c

ACE2

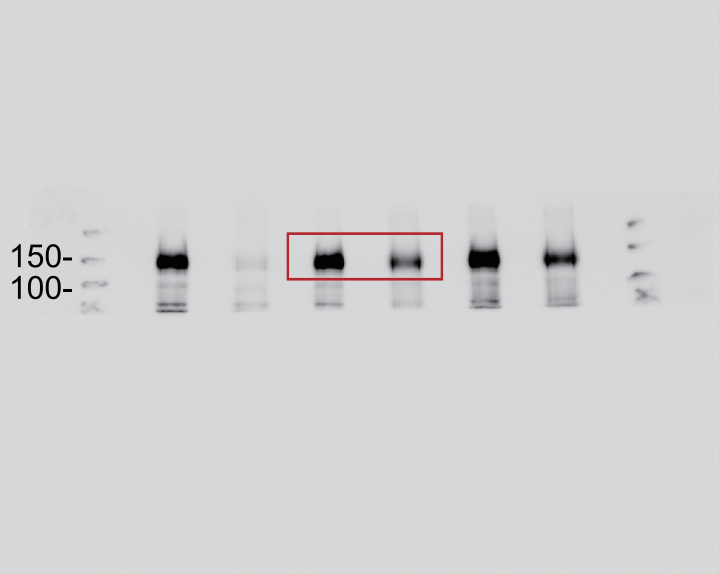

α-actinin

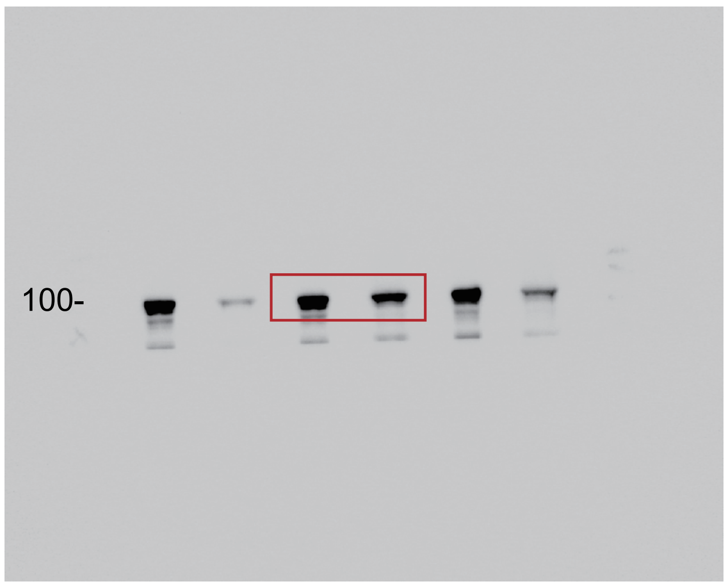

GAPDH

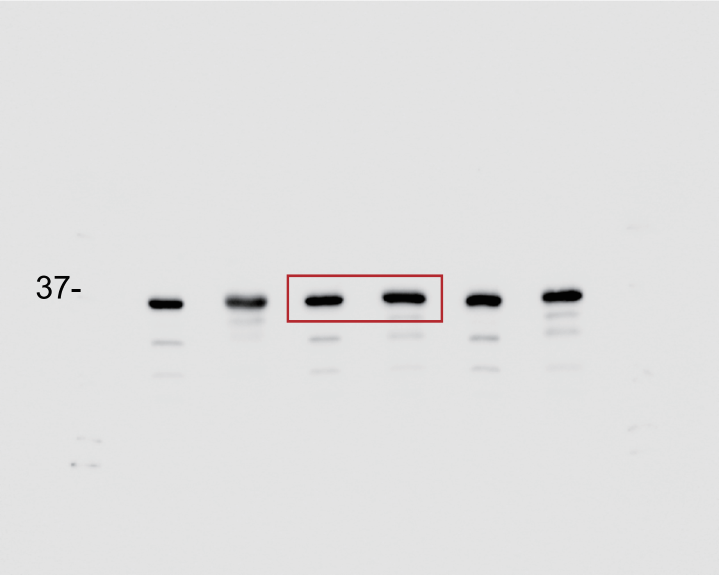

Fig. S1f

ACE2

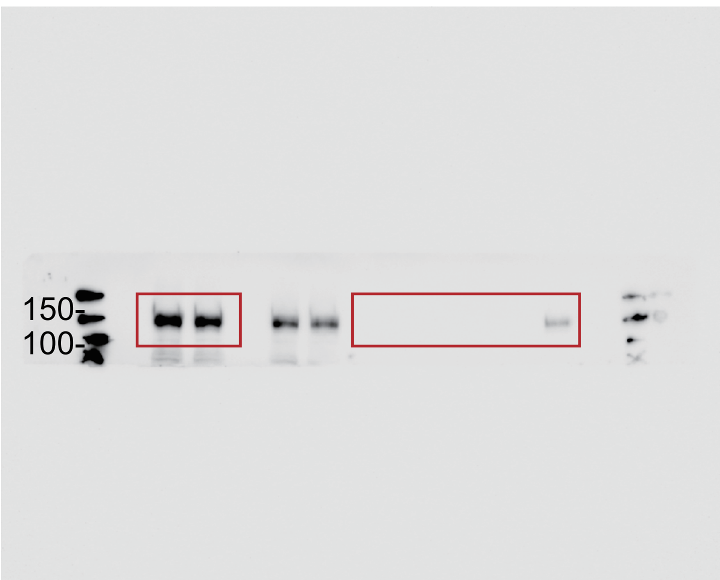

S-RBD

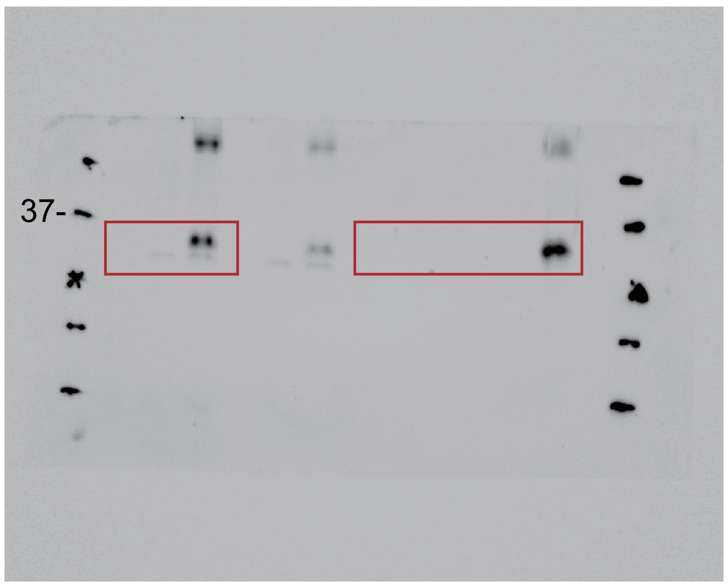

Fig. S1k

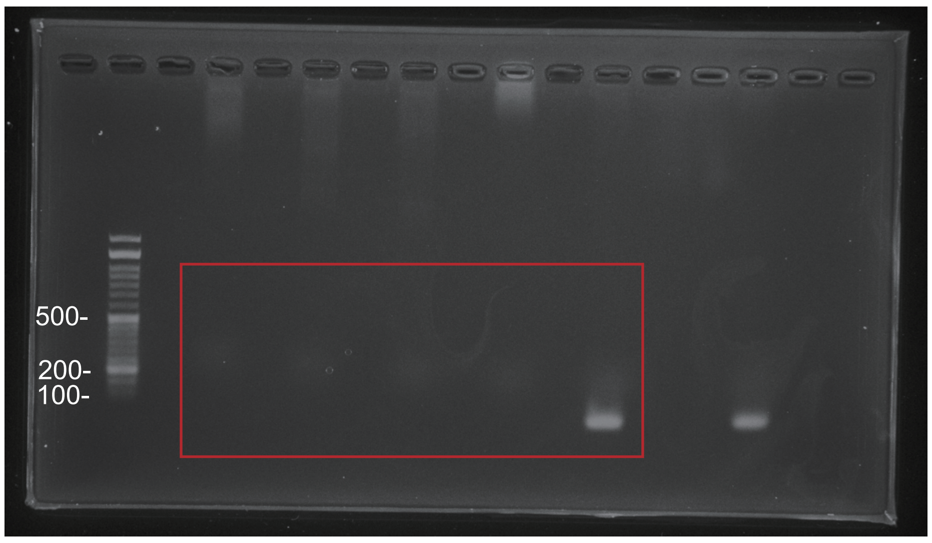

Fig. S2a

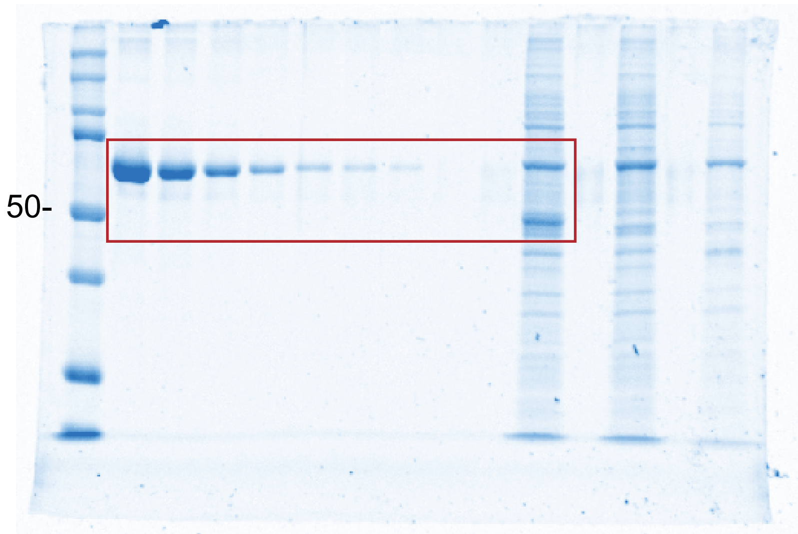

Fig. S2b

S-RBD

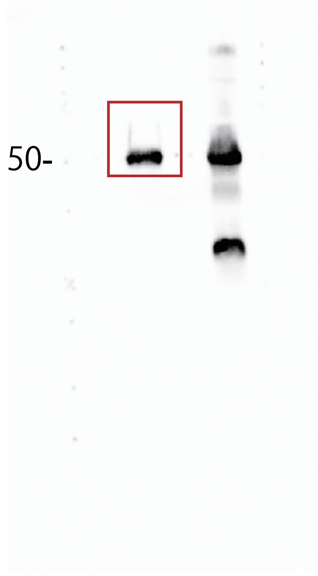

GFP

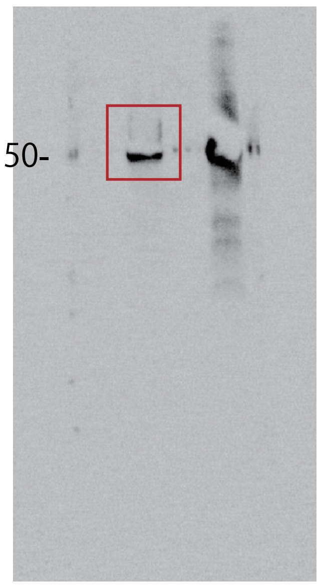

Fig. S2g

ACE2

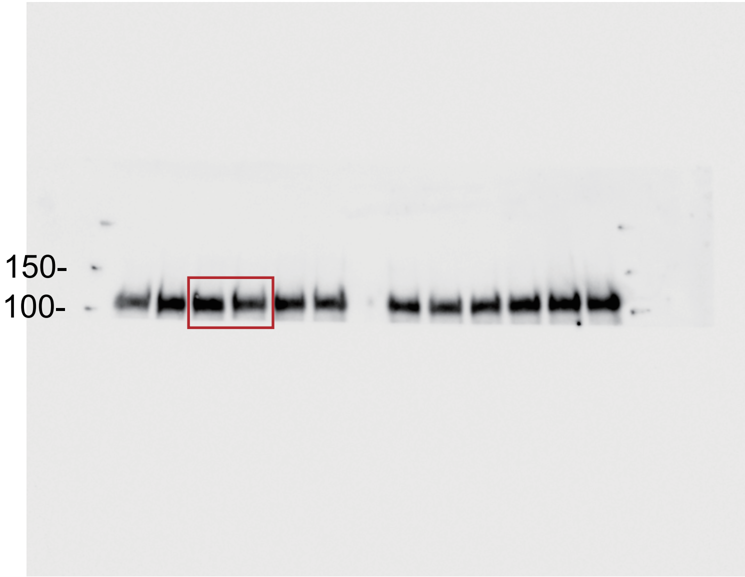

S-RBD

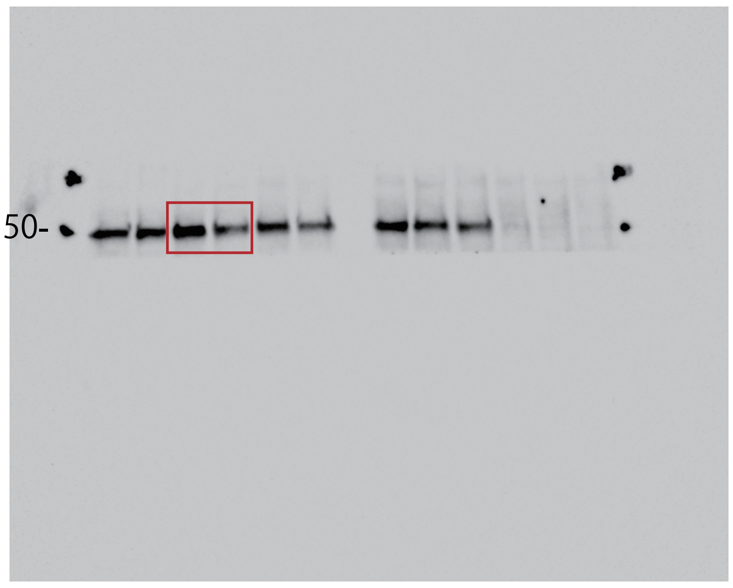

Fig. S2g

GAPDH

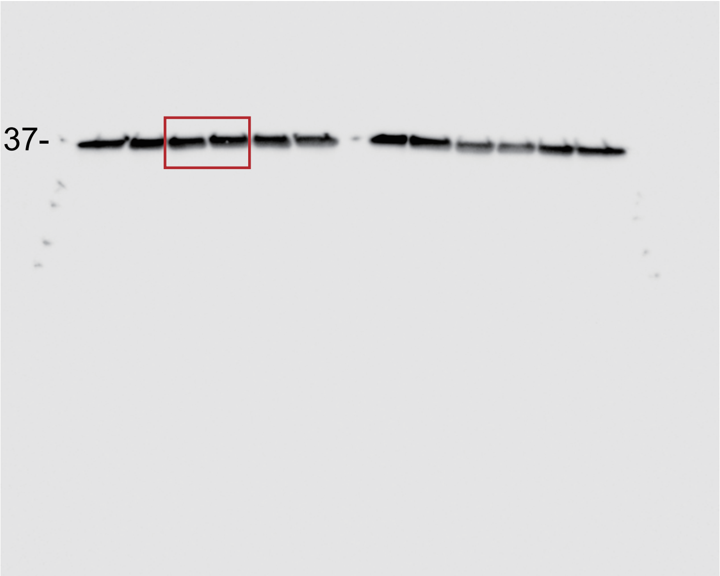

Fig. S4c

ISG15

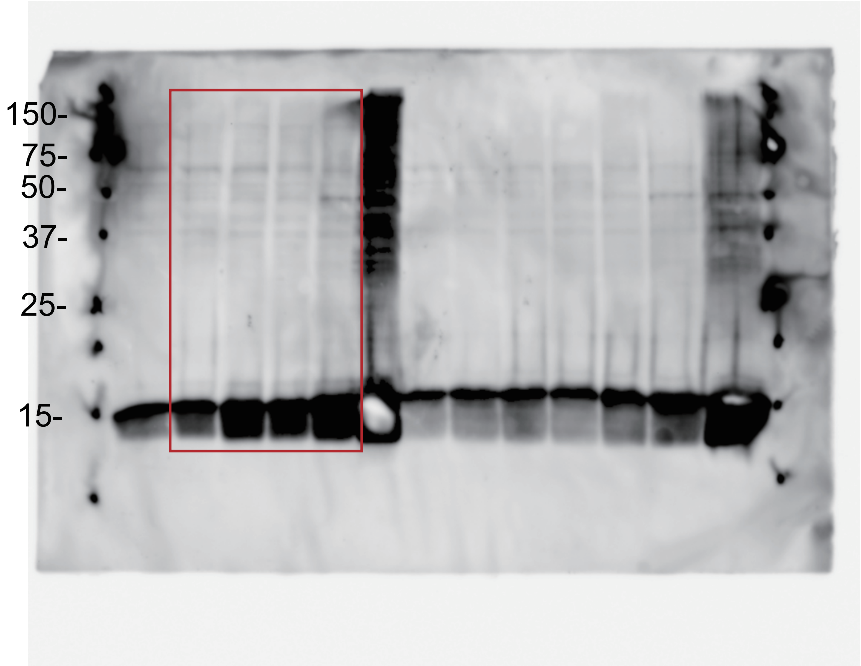

Supplement: Supplementary file 1 — Supplementary Figures. [file 41598_2023_48084_MOESM1_ESM.pdf]
