# Supplementary material for: SARS-CoV-2 spike receptor-binding domain is internalized and promotes protein ISGylation in human induced pluripotent stem cell-derived cardiomyocytes
Source: Sci Rep. 2023 Dec 4;13:21397. doi: 10.1038/s41598-023-48084-7 (PMC10696029; doi:10.1038/s41598-023-48084-7)
Supplement: Supplementary file 2 — Supplementary Information. [file 41598_2023_48084_MOESM2_ESM.docx]

**Supplementary Information**

**SARS-CoV-2 spike receptor-binding domain is internalized and promotes protein ISGylation in human induced pluripotent stem cell-derived cardiomyocytes**

**Author names and affiliations:**

Shota Okuno ^a^, Shuichiro Higo ^a, b,*^, Takumi Kondo ^a^, Mikio Shiba ^a^, Satoshi Kameda ^a^ , Hiroyuki Inoue ^a^, Tomoka Tabata ^a^, Shou Ogawa ^a^, Yu Morishita ^a^, Congcong Sun ^a^, Saki Ishino ^c^, Tomoyuki Honda ^d, e^, Shigeru Miyagawa ^f^, Yasushi Sakata ^a^

**Departments and institutions of origin:**

^a^ Department of Cardiovascular Medicine, Osaka University Graduate School of Medicine, Suita, Osaka, 565-0871, Japan

^b^ Department of Medical Therapeutics for Heart Failure, Osaka University Graduate School of Medicine, Suita, Osaka, 565-0871, Japan

^c^ CoMIT Omics Center, Osaka University Graduate School of Medicine, Suita, Osaka, 565-0871, Japan

^d^ Department of Virology, Okayama University Graduate School of Medicine, Dentistry and Pharmaceutical Sciences, Kita-ku, Okayama, 700-8558, Japan.

^e^ Department of Virology, Faculty of Medicine, Dentistry and Pharmaceutical Sciences, Okayama University, Kita-ku, Okayama, 700-8558, Japan.

^f^ Department of Cardiovascular Surgery, Osaka University Graduate School of Medicine, Suita, Osaka 565-0871, Japan

***Corresponding Author:**

Shuichiro Higo

Specially Appointed Associate Professor

Department of Medical Therapeutics for Heart Failure, Osaka University Graduate School of Medicine

Address: 2-2 Yamadaoka, Suita, Osaka 565-0871, Japan

Tel: +81-6-6210-8260, Fax: +81-6-6210-8261,

E-mail: [higo-s@cardiology.med.osaka-u.ac.jp](mailto:higo-s@cardiology.med.osaka-u.ac.jp)

**Supplementary Methods**

**Reagents and antibodies.**

Reagents and antibodies used in this study are as follows:

Oct-3/4 (C-10) (Santa Cruz Biotechnology, Dallas, Texas, USA, Cat# sc-5279, RRID:AB_628051, x500 dilution), TRA-1-60 (Merck Millipore, Burlington, Massachusetts, USA, Cat# MAB4360, RRID:AB_2119183, x400 dilution), SSEA-4 (Merck Millipore Cat# MAB4304, RRID:AB_177629, x200 dilution), Nanog (Abcam, Cambridge, Massachusetts, USA, Cat# ab80892, RRID:AB_2150114, x200 dilution), ACE2 (Abcam Cat# ab15348, RRID:AB_301861, x500 dilution [immunostaining], x2000 dilution [western blot]), Troponin T (Abcam Cat# ab64623, RRID:AB_1139590, x1000 dilution [immunostaining]), Sarcomeric Alpha Actinin (EA-53) (Abcam Cat# ab9465, RRID:AB_307264, x2000 dilution [western blot]), GAPDH (Santa Cruz Biotechnology Cat# sc-47724, RRID:AB_627678, x2000 dilution [western blot]), His-tag (MEDICAL & BIOLOGICAL LABORATORIES, Minato, Tokyo, Japan, Cat# D291-3, RRID:AB_10597733, x1000 dilution [immunostaining], immunoprecipitation), SARS-CoV-2 Spike RBD (GeneTex, Irvine, California, USA, Cat# GTX635692, RRID:AB_2888564, x2000 dilution [western blot]), Rab5 (Santa Cruz Biotechnology Cat# sc-46692, RRID:AB_628191, x500 dilution [immunostaining], x2000 dilution [western blot]), GFP (MEDICAL & BIOLOGICAL LABORATORIES Cat# PM598-7, RRID:AB_10597267, x2000 dilution [western blot]), ISG15 (Proteintech, Rosemont, Illinois, USA, Cat# 15981-1-AP, RRID:AB_2126302, x2000 dilution [western blot]), normal mouse IgG (Santa Cruz Biotechnology Cat# sc-2025, RRID:AB_737182, immunoprecipitation), Puromycin dihydrochloride (Sigma-Aldrich, Cat# P9620-10ML), Biotinylated SARS-CoV-2 S protein RBD, His, Avitag (ACROBiosystems, Newark, Delaware, USA, Cat# SPD-C82E9), Endosomal maker kit antibody sample kit (Cell Signaling Technology, Danvers, Massachusetts, USA, Cat#12666), Cell Navigator Lysosome Staining dye (AAT Bioquest, Pleasanton, California, USA, Cat# 22652), HumanKine® recombinant human IFN alpha 2A protein (Proteintech, Cat# HZ-1066), Dynabeads Protein G Immunoprecipitation Kit (Thermo Fisher Scientific, Waltham, Massachusetts, USA), Dynasore (Abcam, Cat# ab120192)

**Cell culture and cardiomyocyte differentiation.**

HEK293T cells were maintained in high-glucose Dulbecco’s modified Eagle’s medium (DMEM, Gibco) supplemented with 10% fetal bovine serum (FBS, Gibco), penicillin, streptomycin, and glutamine (PSG, Gibco). The iPSCs were generated from the peripheral blood mononuclear cells as previously described ^1^ and were differentiated into the iPSC-CMs using a chemically defined protocol as previously described ^2^. In this study used the iPSCs generated from a male HCM patient carrying a heterozygous in-frame deletion mutation (c.478_480del, p.Δ160E) in *TNNT2* and the isogenic control with the homozygously corrected alleles using genome editing ^1^. iPSCs generated from a female healthy donor was prepared as control. The culture medium was replaced with the RPMI 1640 medium (Thermo Fisher Scientific) containing recombinant human albumin (Sigma-Aldrich) and L-ascorbic acid 2-phosphate (Sigma-Aldrich) for differentiation. The iPSCs were treated with CHIR99021 (LC Laboratories) (days 0–2), Wnt-C59 (Selleck Chemicals), or XAV-939 (Cayman) (days 2–4). Differentiated monolayer cardiomyocytes were cultured in Roswell RPMI medium until replated for further analysis. Spontaneous beating was observed on day 10 after the induction of differentiation. When replated, the iPSC-CMs were dissociated with 0.25% Trypsin-EDTA (Gibco); suspended with DMEM containing 10% FBS, PSG, and 10 μM Y-27632 (Wako); filtered with 100 μm cell strainer (FALCON); and replated into 12-well, 24-well plates (IWAKI), or 96-well μClear plates (Greiner) precoated with gelatin (Nitta Gelatin) and incubated with DMEM containing serum. The efficiency of differentiation, as evaluated by flow cytometry using an anti-TnT antibody, was approximately 90%. The iPSC-CMs expressed the standard cardiac sarcomeric marker, α-actinin.

**Immunofluorescent staining.**

The iPSCs were seeded into 96-well μClear plates at 1,000 cells/well and incubated at 37ºC for colony formation. Differentiated iPSC-CMs were seeded at 1-2 x 10^4^ cells/well in 96-well μClear plates precoated with gelatin. For immunostaining, cells were fixed with 4% paraformaldehyde for 15 min, permeabilized with 0.5% Triton X-100 for 15 min, and blocked with 1% bovine serum albumin (BSA) at room temperature for 30 min or at 4°C for overnight. Primary antibodies were diluted by 1% BSA, added to each well, and incubated for 1 h at room temperature or overnight at 4°C. Secondary antibodies conjugated with Alexa Fluor Dyes (Molecular Probe) including Hoechst 33342 for nuclear staining were added and incubated at room temperature for 30 min. All images were acquired using the IN Cell Analyzer 6000 (GE Healthcare).

**Western Blotting.**

The cells were washed with cold Dulbecco’s phosphate-buffered saline (PBS) and directly lysed with SDS buffer (10% SDS, 50 mM Tris-HCl (pH7.4), and 5 mM EDTA). Protein concentration was determined using a BCA Protein Assay Kit (Thermo Fisher Scientific). Lysate samples were mixed with 4 × Laemmli sample buffer (Bio-Rad) and mercaptoethanol (2.5%). Proteins were separated by sodium dodecyl sulfate-polyacrylamide gel electrophoresis and transferred to PVDF membranes. The antibodies were diluted in 3% nonfat milk. After blocking with 3% nonfat milk for 1 h, the transferred membrane was incubated with primary antibody at 4°C for overnight and with secondary antibody at room temperature for 30 min. The membrane signals were detected by chemiluminescence using ECL or ECL prime reagent (GE Healthcare). The protein expression levels were quantified using ImageQuant TL (GE Healthcare). The expression level of each protein was normalized to that of GAPDH.

**Immunoprecipitation.**

Immunoprecipitation using ACE2-WT-iPSC-CMs treated with or without His-tagged S-RBD for 48 h was performed using Dynabeads Protein G (Thermo Fisher Scientific), according to the manufacturer’s instructions. Six micrograms of anti-His-tag antibodies or mouse IgG control (as negative control) were conjugated to protein G beads for 30 min at 4°C. Beads were washed once in TNE buffer (10 mM Tris-HCl, pH 7.2, 100 mM NaCl, 1mM EDTA, 1% CHAPS) and incubated with the lysate samples in TNE buffer for 4 hours at 4°C. After washing three times in TNE buffer, beads were eluted with Elusion Buffer (Thermo Fisher) and SDS sample buffer at 95°C for 3 min. The eluted samples were analyzed by SDS-PAGE followed by western blotting using an anti-ACE2 antibody and SARS-CoV-2 Spike RBD antibody.

**Plasmid construction.**

The gRNA sequences targeting the genomic region surrounding exon 2 of ACE2 were designed using the CRISPR Design Tool ^3^ and cloned into the pX459 vector, as previously described ^4^. The full-length human RAB5A coding sequence was cloned from human cDNA using specific PCR primers and subcloned into the pENTR/D-TOPO vector (Thermo Fisher Scientific). To generate the N-terminal DsRed fluorescent protein, a DsRed epitope was inserted before the coding sequence. For adeno-associated virus (AAV) generation, the N-terminal DsRed-fused full-length RAB5A sequence was subcloned into the pAAV vector (TaKaRa). pcDNA3-SARS-CoV-2-S-RBD-sfGFP (Addgene plasmid # 141184) ^5^ was used to generate recombinant S-RBD-sfGFP.

**Generation of isogenic ACE2-KO-iPSC-CMs using CRISPR/Cas9 genome editing.**

Five micrograms of pX459 vector encoding gRNA against *ACE2* sequence to introduce non-homologous end joining (NHEJ) in *ACE2* was electroporated into 3 × 10^5^ cells using a NEPA 21 electroporator (poring pulse: pulse voltage 125 V, pulse width 5 ms, pulse interval 50 ms, pulse number 2. Transfer pulse: pulse voltage, 20 V; pulse width, 20 ms; pulse interval, 50 ms; pulse number, 5). Puromycin (0.3 μg/mL) was added on the day after electroporation. Thirty-six hours after the addition of puromycin, the culture medium was replaced with puromycin-free medium. Seven days later, the iPSCs were passaged in 6-well plates at a density of 200-400 cells/well for clonal colony formation. Genomic DNA was extracted using a QIAamp DNA Mini Kit (QIAGEN). Target regions were amplified by PCR (KOD FX Neo, TOYOBO) as follows: 94°C for 2 min, followed by 33 cycles of 98°C for 10 s, annealing temperature (depending on primer sequences) for 30 s and 68°C for 30 s. Primer sequences are listed in Supplemental Table 1. After purifying the PCR products using the QIAquick PCR Purification Kit (QIAGEN), the genome editing results were evaluated by Sanger sequencing. NHEJ clones were detected in the iPSCs transfected with pX459 encoding gRNA. After iPSC colony formation, each colony (at least 24 colonies) transfected with pX459 encoding gRNA was picked and dissociated into single cells in a sterile tube. The selected colonies were split into two 96-well plates for genotyping and cell expansion. The target genomic region was amplified using PCR and evaluated using Sanger sequencing.

**Generation of AAV and transduction in the iPSC-CMs.**

To generate AAV2, HEK293T cells were transfected with a pAAV vector encoding N-terminally DsRed-fused RAB5A, pHelper vector, and pRC2-mi342 vector (AAVpro Helper Free System, TaKaRa) using calcium phosphate transfection (CalPhos Mammalian Transfection Kit,TaKaRa). Seventy-two hours after transfection, HEK293T cells were detached by the addition of 1/80 volume of 0.5M EDTA (pH 8.0), and then pelleted via low-speed centrifugation (2000 × g for 10 min). Cell pellets were lysed with AAV Extraction Solution A and centrifuged (9000 x g for 10 min). AAV Extraction Solution B was added to the collected supernatant and stored at -80°C. AAV generated from HEK293T cells was purified using the AAVpro Purification Kit (TaKaRa), and the viral titer was calculated using the AAV Titration Kit (TaKaRa). For immunostaining or live cell imaging, the iPSC-CMs around 14 days after differentiation were seeded into 96-well plates (1-2 x 10^4^ cells/well) or glass-based dish (1.2 x 10^5^ cells/well), then transduced with AAV2 at 1.0–2.0 x 10^4^ vg/cell 7 days after replating.

**Live cell imaging and confocal microscopy.**

Differentiated iPSC-CMs were seeded at 0.8-1.2 × 10^5^ cells/well into a glass-based dish precoated with gelatin, then transduced with AAV2-DsRed-RAB5A at 1.0–2.0 × 10^4^ vg/cell 7 days later. The iPSC-CMs were then treated with 750 ng/mL S-RBD-sfGFP immediately prior to observation. Images were acquired using a spinning disk-based confocal microscope (IXplore SpinSR; Olympus). For live cell imaging of lysosomes, the iPSC-CMs were stained with the Cell Navigator Lysosome Staining dye for 30 min, and the excess stain was washed away 1 h before observation.

**RNA extraction, quantitative real-time PCR, and human NF-kβ pathway PCR array.**

The iPSC-CMs were replated at 1.2 x 10^5^ cells/well into 24-well plates on day 14 after differentiation and treated with S-RBD-sfGFP and control-GFP at two different doses: 600 ng/mL S-RBD-sfGFP (low) and 1,800 ng/mL S-RBD-sfGFP (high) for 48 h on day 26-28. Total RNA was extracted using the RNeasy mini kit (QIAGEN) and converted to cDNA using a high-capacity RNA-to-cDNA RT kit (Thermo Fisher Scientific). Quantitative real-time PCR was performed using SYBR green assay or TaqMan assay (THUNDERBIRD SYBR qPCR Mix, THUNDERBIRD probe qPCR mix, TOYOBO). The PCR primers and probes used for quantitative real-time PCR are listed in Supplemental Table 1. The cocktail for PCR array was prepared by adding master mix and nuclease-free water to the diluted cDNA samples, and 20 μL of this cocktail was added to each well of the 96-well PCR array plate (Taqman^TM^ Array Human NFKB Pathway, Thermo Fisher Scientific) containing primers and probes for the 92 genes in human NF-kβ pathway and five housekeeping control genes (Supplemental Table 2). All samples were processed in duplicate. The level of each transcript was quantified using the threshold cycle (Ct) method, with GAPDH as an internal control. After amplification, real-time PCR data acquisition and analysis were performed using QuantStudio Real-Time PCR Software or ExpressionSuite Software v1.3 (Thermo Fisher Scientific).

**Electrical stimulation protocol.**

On day 11, iPSC-CMs were replated into a 6-well plate precoated with gelatin and incubated with DMEM containing 10% FBS, PSG. On day 14, we then initiated electrical stimulation at 2 Hz, with an electric field of 7 V/cm, and with 5 ms pulse (C-Pace EM, IonOptix) and continued it for 1 week. After that iPSC-CMs were replated into gelatin-coated 24-well plates on day 21 and incubated with DMEM containing serum for further analysis.

**Supplementary Figure Legends**

**Supplementary Figure S1. ACE2 protein expression in iPSC-CMs, binding of SARS-CoV-2 S-RBD and ACE2 and generation of isogenic *ACE2*-KO-iPSCs.**

1. Whole cell lysates were extracted from the iPSC-CMs with HCM mutation and the isogenic iPSC-CMs with the corrected HCM mutation on day 21-28 after differentiation and analyzed by western blotting using the indicated antibodies. Original blots are presented in Supplementary Fig. S7.
2. Quantified ACE2 protein expression levels were normalized by GAPDH expression (n = 3). Data are presented as the mean ± SD. Statistical differences were calculated using Student’s t-test.
3. Whole cell lysates were extracted from the iPSC-CMs with the corrected HCM mutation and the iPSC-CMs from a female healthy donor on day 21-28 after differentiation and analyzed by western blotting using the indicated antibodies. Original blots are presented in Supplementary Fig. S7.
4. Quantified ACE2 protein expression levels were normalized by GAPDH expression (n = 3). Data are presented as the mean ± SD. Statistical differences were calculated using Student’s t-test.
5. *ACE2*-WT-iPSC-CMs incubated with or without 1,250 ng/mL purified His-tagged SARS-CoV-2 S-RBD protein for 48 h were immunostained on day 28 after differentiation with the indicated antibodies. Peripheral accumulation of S-RBD was observed in the iPSC-CMs treated with S-RBD, while non-specific nuclear staining was observed in both images.
6. Protein extracts from *ACE2*-WT-iPSC-CMs treated with or without His-tagged S-RBD was incubated with anti-His-tag antibodies or mouse IgG control conjugated to protein G magnetic beads. After immunoprecipitation the eluted samples were analyzed by western blotting using the indicated antibodies. Original blots are presented in Supplementary Fig. S8.
7. Targeted site of genome editing in exon 2 of human ACE2.
   The gRNA used an AGG as a PAM sequence.
8. Direct Sanger sequence analysis of the ACE2 locus using genomic DNA obtained from isogenic *ACE2*-WT-iPSCs and *ACE2*-KO-iPSCs.
9. Bright-field images of isogenic *ACE2*-WT and *ACE2*-KO iPSC clones.
10. Isogenic *ACE2*-KO-iPSCs were cultured in 96-well plates, fixed, and immunostained with the indicated antibodies. Nuclei were stained with Hoechst.
11. PCR was performed to detect the transgenes generated from the residual Sendai viral vectors using cDNA obtained from the generated *ACE2*-KO-iPSCs. The primers used for PCR analysis are listed in Supplemental Table 1. The original gel is presented in Supplementary Fig. S8.
12. Karyotype analysis of the generated *ACE2*-KO-iPSCs.

**Supplementary Figure S2. Live cell imaging using iPSC-CMs treated with recombinant S-RBD-sfGFP.**

1. Recombinant S-RBD-sfGFP was analyzed using Coomassie Brilliant Blue Staining. A band indicating S-RBD-sfGFP was detected (white arrow). The concentration of S-RBD-sfGFP can be estimated to be 5 ng/μL by comparing with BSA control. The original blot is presented in Supplementary Fig. S8.
2. The presence of recombinant S-RBD-sfGFP was confirmed by western blotting using the indicated antibodies. Original blots are presented in Supplementary Fig. S8.
3. Time course of live cell imaging after replating of the iPSC-CMs. Differentiated cardiomyocytes were replated on day 14 after differentiation and incubated with SARS-CoV-2 S-RBD-sfGFP for 48 h before live cell imaging.
4. Live cell imaging of *ACE2*-WT and *ACE2*-KO iPSC-CMs following S-RBD-sfGFP treatment. GFP-positive cells were observed in *ACE2-*WT-iPSC-CMs in a dose-dependent manner, but not in *ACE2*-KO-iPSC-CMs after treatment with the generated S-RBD-sfGFP for 48 h.
5. The image of line profiles for quantification of the relative intensity variation of S-RBD-sfGFP signals. Line profile 1 was within the GFP positive iPSC-CM and Line profile 2 set as the background.
6. The immunofluorescent intensities obtained from the line profile within the GFP-positive iPSC-CMs (Line profile 1) (*F*) and from the background (Line profile 2) (F_0_) were measured in each time point. Relative intensities calculated as (F − F_0_)/F_0_ were plotted.
7. Whole cell lysates were extracted from iPSC-CMs on day 28 after differentiation and analyzed by western blotting using the indicated antibodies. After pre-treatment with dynamin inhibitor (Dynasore; 80 μM) or DMSO for 30 min, iPSC-CMs were additionally treated with 1,200 ng/mL SARS-CoV-2 S-RBD-sfGFP and incubated for 12 h prior to western blotting. Original blots are presented in Supplementary Fig. S8, S9.
8. Quantified S-RBD-sfGFP expression levels were normalized by GAPDH expression in iPSC-CMs treated with dynamin inhibitor or DMSO (n = 3). Data are presented as the mean ± SD. Statistical differences were calculated using the Student’s t-test. **p <0.01.

**Supplementary Figure S3. Violin plots of NF-kβ pathway-related gene in non-cardiomyocytes and IFN-responsive genes in matured cardiomyocytes evaluated by scRNA-seq.**

1. Violin plots showing normalized transcript abundance of *CXCL1* from pairwise cluster comparisons between non-cardiomyocytes treated with control-GFP and S-RBD-sfGFP by scRNA-seq

Boxplots are presented as medians and interquartile ranges, and whiskers represent the 5^th^ and 95^th^ percentiles.

1. Violin plots showing normalized transcript abundance of the top five upregulated genes from the pairwise cluster comparison between mature cardiomyocytes treated with control-GFP and S-RBD-sfGFP by scRNA-seq

Boxplots are presented as medians and interquartile ranges, and whiskers represent the 5^th^ and 95^th^ percentiles.

**Supplementary Figure S4. Validation of upregulation of IFN-responsive genes in matured cardiomyocytes with electrical stimulation, violin plots of E3 ligase genes in matured cardiomyocytes and increased protein ISGylation in the iPSC-CMs after S-RBD treatment.**

1. *ACE2*-WT iPSC-CMs were electrically stimulated for 1 week to promote maturation and treated with 1,800 ng/mL S-RBD-sfGFP for 48 h. The mRNA expression levels of *IFI6, ISG15, IFITM3, SOCS3,* and *USP18* were normalized by *GAPDH* expression and validated using quantitative real-time PCR. Relative expression levels were normalized to the expression levels in each iPSC-CM treated with control GFP (n = 10 independent biological replicates). Data are presented as medians and interquartile ranges. Statistical differences were calculated using the Mann–Whitney U test. **p <0.01, ***p <0.001.
2. Violin plots showing normalized transcript abundance of E3 ligase genes from pairwise cluster comparison between mature cardiomyocytes treated with control-GFP and S-RBD-sfGFP by scRNA-seq

Boxplots are presented as medians and interquartile ranges, and whiskers represent the 5^th^ and 95^th^ percentiles.

1. Whole-cell lysates were extracted from *ACE2*-WT-iPSC-CMs on day 28 after differentiation and analyzed by western blotting using the indicated antibodies. *ACE2*-WT-iPSC-CMs were incubated with 1,200 ng/mL (duplicate), 6,000 ng/mL SARS-CoV-2 S-RBD-sfGFP, or control GFP for 48 h before western blotting. Original blots are presented in Supplementary Fig. S9.

**Supplementary Video. Sequential observation of endocytosis of SARS-CoV-2 S-RBD-sfGFP in *ACE2*-WT-iPSC-CMs transduced with AAV2-DsRed-RAB5A.**

Images were captured every 5 min for 50 min. White circle ROIs show the particles of S-RBD-sfGFP co-localized with DsRed-RAB5A.

**Supplemental References**

1 Kondo, T. *et al.* Human-Induced Pluripotent Stem Cell-Derived Cardiomyocyte Model for TNNT2 Delta160E-Induced Cardiomyopathy. *Circ Genom Precis Med* **15**, e003522, doi:10.1161/CIRCGEN.121.003522 (2022).

2 Burridge, P. W. *et al.* Chemically defined generation of human cardiomyocytes. *Nat Methods* **11**, 855-860, doi:10.1038/nmeth.2999 (2014).

3 Hsu, P. D. *et al.* DNA targeting specificity of RNA-guided Cas9 nucleases. *Nat Biotechnol* **31**, 827-832, doi:10.1038/nbt.2647 (2013).

4 Ran, F. A. *et al.* Genome engineering using the CRISPR-Cas9 system. *Nat Protoc* **8**, 2281-2308, doi:10.1038/nprot.2013.143 (2013).

5 Chan, K. K. *et al.* Engineering human ACE2 to optimize binding to the spike protein of SARS coronavirus 2. *Science* **369**, 1261-1265, doi:10.1126/science.abc0870 (2020).
